# Supplementary figures and images for: New insights into the all-testis differentiation in zebrafish with compromised endogenous androgen and estrogen synthesis
Source: PLoS Genet. 2024 Mar 7;20(3):e1011170. doi: 10.1371/journal.pgen.1011170 (PMC10919652; doi:10.1371/journal.pgen.1011170)

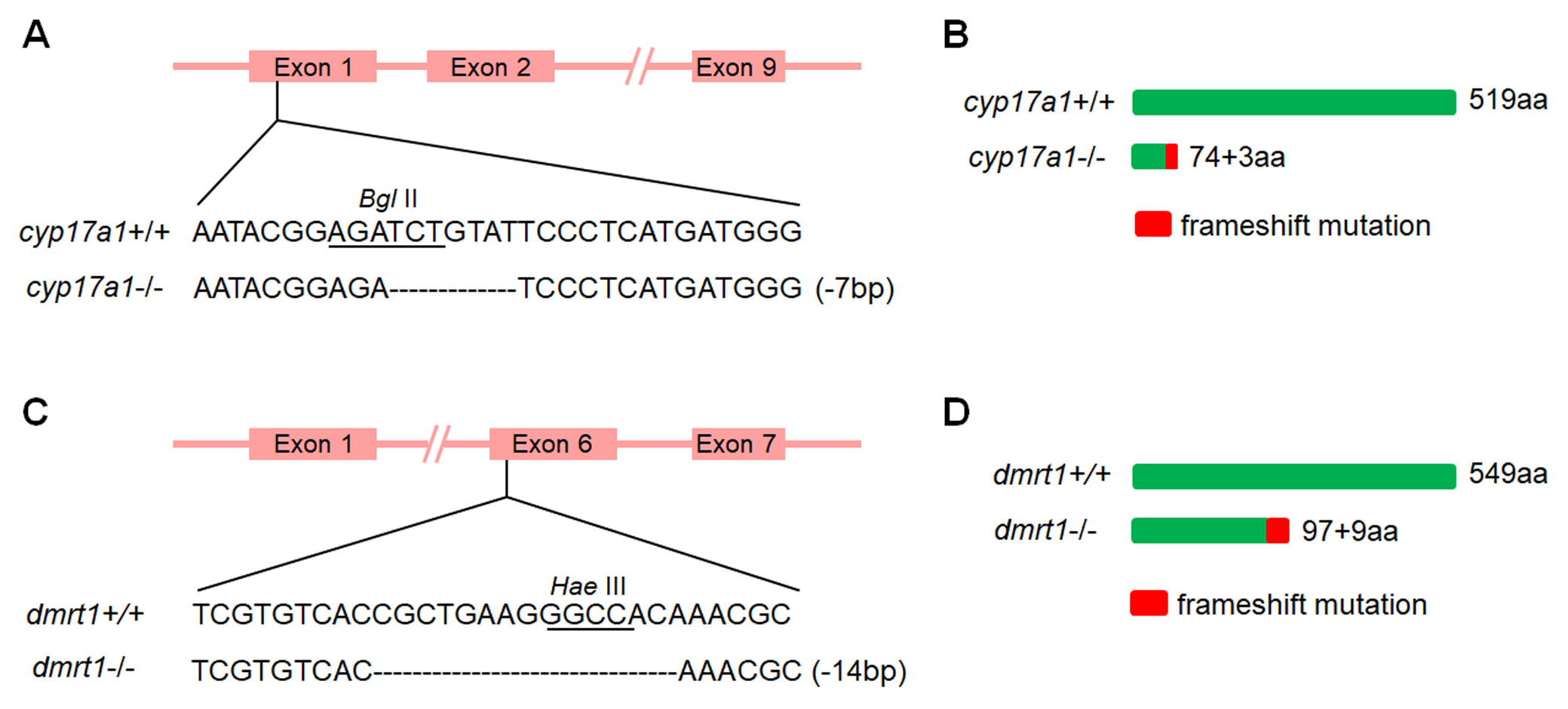

Supplement: S1 Fig — (A) Schematic representation of wildtype (cyp17a1+/+) and the mutant line of cyp17a1 alleles in the first exon. (B) Schematic representation of the putative peptide of wildtype (cyp17a1+/+) and the mutated Cyp17a1 peptides. (C) Schematic representation of wildtype (dmrt1+/+) and the mutant line of dmrt1 alleles in the sixth exon. (D) Schematic representation of the putative peptide of wildtype (dmrt1+/+) and the mutated Dmrt1 peptides. (TIF) [file pgen.1011170.s001.tif]

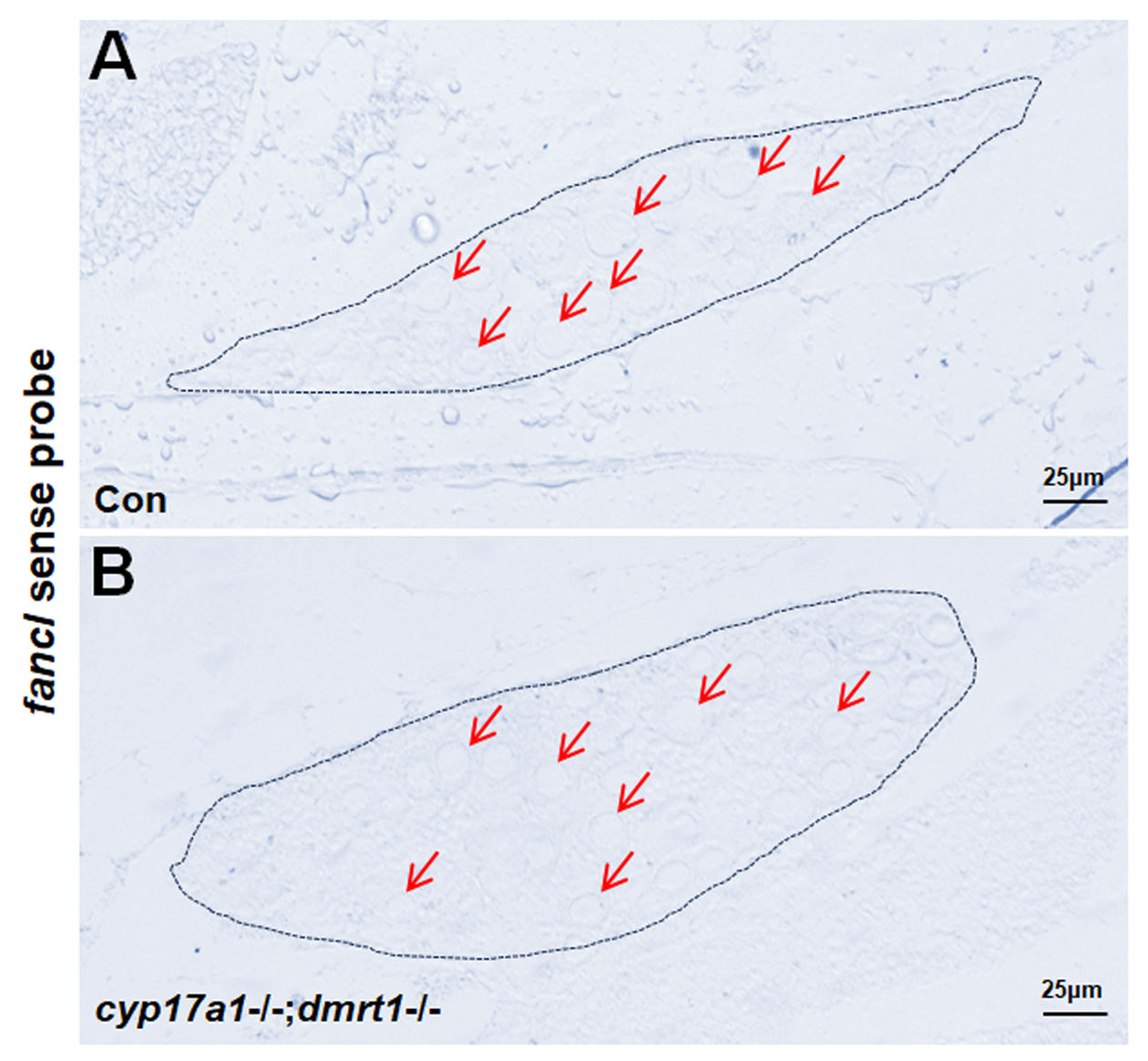

Supplement: S2 Fig — (A) Control fish at 25 dpf. (B) cyp17a1-/-;dmrt1-/- fish at 25 dpf. Arrows point to the immature oocytes. (TIF) [file pgen.1011170.s002.tif]

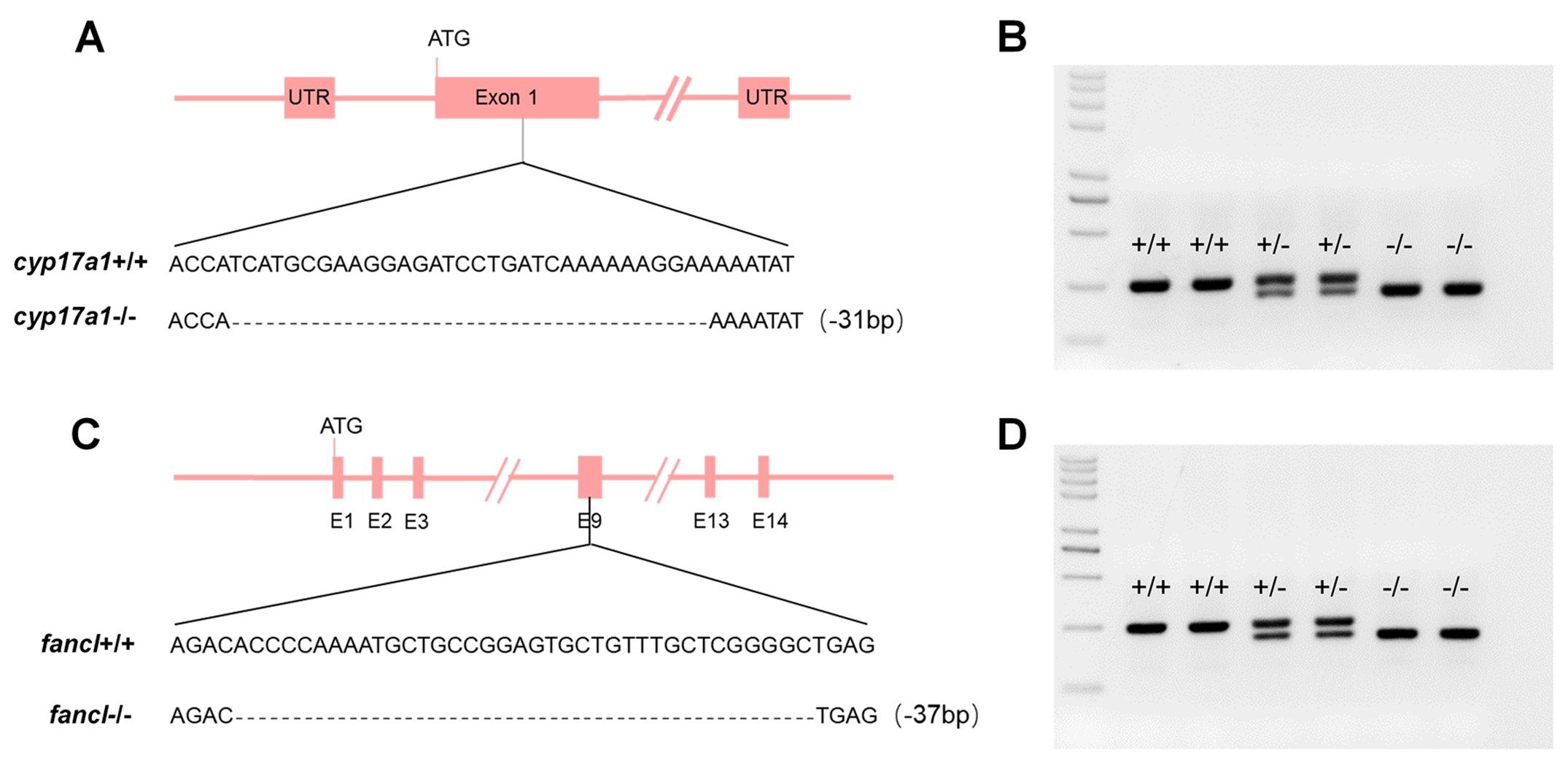

Supplement: S3 Fig — (A) Schematic representation of the genomic locus for target disruption of cyp17a1. UTR, untranslated region. (B) The PCR results using the fish genomic DNA for genotyping cyp17a1, including cyp17a1+/+, cyp17a1+/- and cyp17a1-/-. (C) Schematic representation of genomic locus for target disruption of fancl. E, exon. (D) The PCR results using the fish genomic DNA for genotyping fancl, including fancl+/+, fancl+/- and fancl-/-. (TIF) [file pgen.1011170.s003.tif]

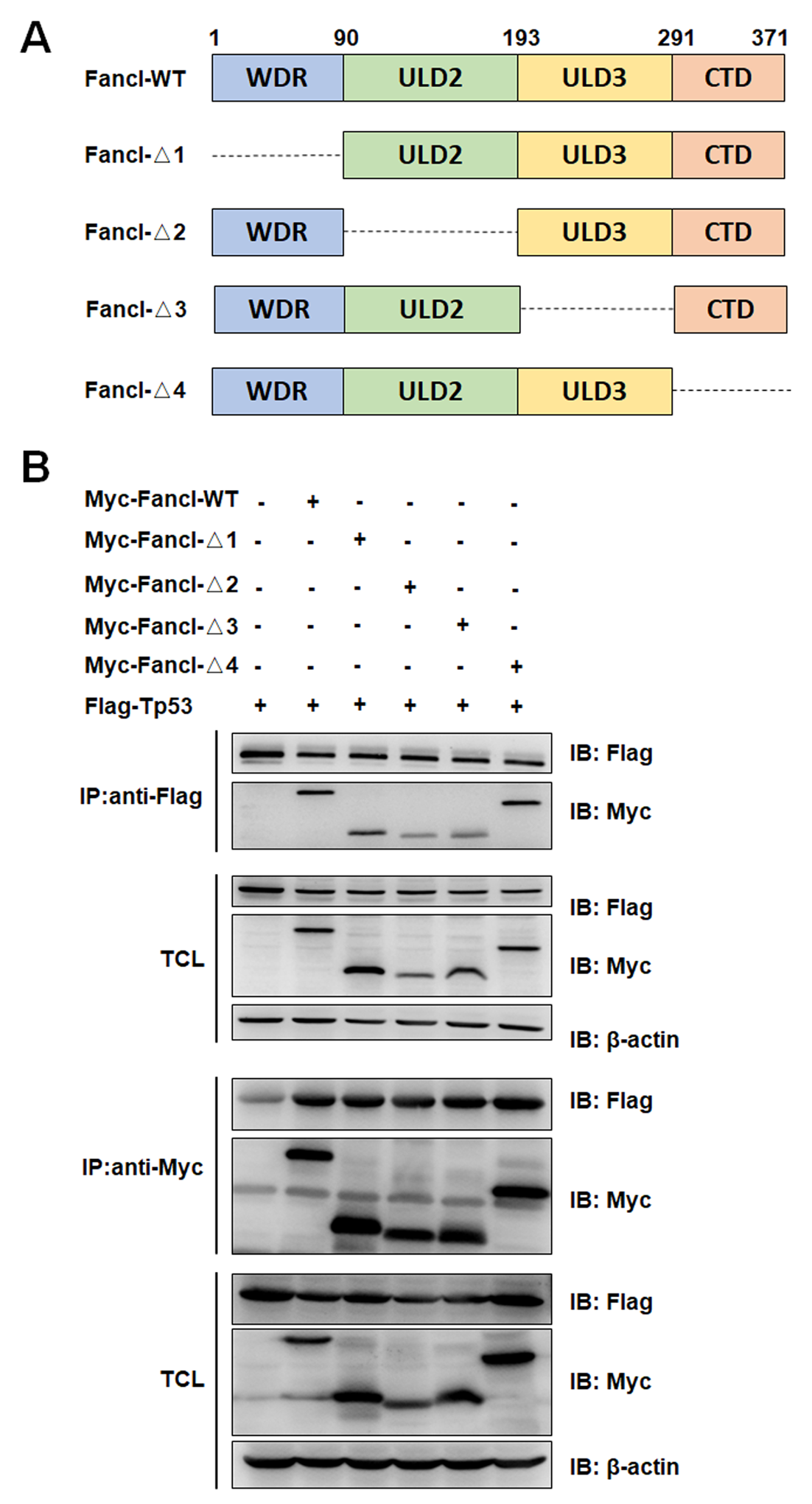

Supplement: S4 Fig — (A) Myc-tagged Fancl with single domain mutation and Flag-tagged TP53 were transfected into HEK293T cells. Both the anti-Myc and anti-Flag antibody-conjugated agarose beads were used for immunoprecipitation. (B) Domain mapping revealed that the single domain mutation of Fancl did not affect its association with TP53. WDR, WD-repeat domain. ULD2, UBC-like domain 2. ULD3, UBC-like domain 3. CTD, C-terminal domain. IP, immunoprecipitation. IB, immunoblotting. TCL, total cell lysate. (TIF) [file pgen.1011170.s004.tif]
