# Supplementary material for: New insights into the all-testis differentiation in zebrafish with compromised endogenous androgen and estrogen synthesis
Source: PLoS Genet. 2024 Mar 7;20(3):e1011170. doi: 10.1371/journal.pgen.1011170 (PMC10919652; doi:10.1371/journal.pgen.1011170)
Supplement: Attachment — Submitted filename: Response letter to P Genet 0104.doc [file pgen.1011170.s011.doc]

Ms. Ref. No.: PGENETICS-D-23-01005

Title: Insights into the all-testis differentiation in zebrafish with compromised endogenous androgen and estrogen synthesis

**Dear Editor of *PLOS Genetics*,**

We appreciate the constructive comments of the reviewers! We have revised the manuscript according to the suggestions and addressed each of the reviewers' criticisms. A detailed point-by-point response to the reviewers' comments is provided. The comments are highlighted in blue, and our responses are given below.

Here, we list the major changes and essential revisions we made to the manuscript:

1. The modified Figure 1. The staging system adopted for ovarian follicles was conducted based on the original definition of Selman et al as modified by the researchers. The estradiol concentration of control female, control male, cyp17a1-/- fish, dmrt1-/- female and cyp17a1-/-;dmrt1-/- fish has been measured. The histological analysis of the gonads of control fish, cyp17a1-/- fish, dmrt1-/- fish and cyp17a1-/-;dmrt1-/- fish at 50 dpf were provided.

2. The modified Figure 2. Previous Figure 3 has been rearranged as Figure 2. Multiple comparisons of fancl expression in control, dmrt1-/- and cyp17a1-/-;dmrt1-/- fish at 17 and 23 dpf were performed using one-way ANOVA followed by Fisher's LSD test. In situ hybridization was repeated on cryosections of presumptive ovaries from control and cyp17a1-/-;dmrt1-/- fish using the antisense probe of fancl and the image was updated. The effect of Dmrt1 and androgen signaling on the regulation of relative luciferase activity driven by the fancl promoter in vitro was determined.

3. The modified Figure 3. The previous Figure 4 has been rearranged as Figure 3. The visualizations of the dissected testis from control male fish, dmrt1-/- male fish, cyp17a1-/-;dmrt1-/-;fancl-/- fish under microscopy after fixing with Bouin's solution were conducted. The anatomical examination and histological analysis of the dmrt1-/-;fancl-/- fish were analyzed and observed according to reviewer’s suggestion.

4. The modified Figure 4. The previous Figure 3 has been separated into current Figure 4 and Figure 5. The assessment of whether the truncations that eliminate multiple domains significantly attenuates the binding of the Fancl to Tp53 in vitro was performed. The multiple domains, WD-repeat domain (WDR) and C-terminal domain (CTD), of Fancl are required for their interaction, rather than single mutant domain.

5. The modified Figure 5. Myc-tagged Fancl decreased the levels of Flag-tagged Tp53 in a dose-dependent manner by increasing the K48-linked ubiquitination of Tp53. Statistical analyses were performed using one-way ANOVA, followed by Fisher’s LSD test.

6. The modified Figure 6. The staging system adopted for ovarian follicles was conducted based on the original definition of Selman et al as modified by the researchers.

7. The new Figure S1. In situ hybridization was performed on cryosections of presumptive ovaries using the sense probe of fancl.

8. The modified Figure S2. The previous Figure S1, which showed the schematic view of the body trunk of zebrafish at 17 and 23 dpf sampled for RNA extraction has been deleted according to reviewer’s suggestion. The previous Figure S4 was rearranged as Figure S2.

9. The modified Figure S3. The association analysis of Fancl with the single domain mutation and Tp53 was updated by combining the previous Figure S2 and Figure 3C and E.

Moreover, the title, abstract, author summary, instructions, results, discussions and materials and methods have been re-worded thoroughly in the revised manuscript. After these changes, we hope that our revised version of the manuscript is now acceptable for publication in your journal. We are also willing to make further revisions and improvements if the additional criticisms are raised by reviewers.

Thank you very much for your consideration!

Zhan Yin

Ph. D.

Institute of Hydrobiology

Chinese Academy of Sciences

## *Response to Reviewer 1:*

**Reviewer #1:**

The factors regulating sex determination and differentiation are not fully understood, even in organisms where dedicated sex chromosomes are present. In zebrafish, domesticated strains have lost the ZW based sex determination that is found in wild strains and as in other animals, the mechanisms of sex determination and sex-specific differentiation are not understood. Despite differences in upstream activators or triggers, differentiation of primary and secondary sex traits converges on regulation of androgen and estrogen levels. Remarkably, prior work in zebrafish established that normal spermatogenesis could occur in the absence androgen receptor or the Cytochrome P450, Cyp17a1a, when supplemented with progestin. In this work the authors investigated the relationship between a conserved male differentiation factor, Dmrt1, and Cyp17a1a, a key enzyme in androgen and estrogen synthesis. The authors took a genetic approach to examine the relationship between these factors and sex determination and differentiation and found that in contrast to loss of Cyp17a1a, which causes testis only development in zebrafish, loss of Dmrt1 in cyp17a1a mutants resulted in development of ovaries with only early-stage oocytes. They provide evidence that supplementing dmrt1;cyp17a1a mutants with estradiol supported oocyte development to later stages. Based on these observations the authors conclude that Dmrtt1 is required for testis differentiation and that estradiol is not required for ovary differentiation but is important for ovary development and maturation. Using an RNAseq approach, the authors determined that fancl expression was higher in dmrt1;cyp17a1a double mutants. Based on its elevated expression and because prior work in the field implicated Fanc family members in ovary development, and germ cell loss in ovary to testis transformation, the authors generated triple mutants lacking Dmrt1, Cyp17a1a, and Fancl. The authors found that these triple mutants, like cyp17a1a and fancl single mutants, developed exclusively as males, but unlike the fancl or cyp17a1a single mutants, the triple mutants had hypoplastic and abnormal testis development due to lack of Dmrt1. Tunel and germ cell analyses showed abnormal germ cell development and cell death in triple mutant testis. Since Tp53 was previously shown to suppress the loss of oocytes and sex reversal previously associated with mutations of Fancl family members, the authors investigated a potential role for Fancl in repressing Tp53. The authors provide evidence that Fancl and Tp53 bind to one another in 293 cells and identified the TMD domain as Tp53 as important for that interaction. Similar attempts to map the interaction domain of Fancl suggest that multiple domains may be involved and are sufficient for interaction with Tp53 in 293 cells. The authors performed in vitro assays in 293 cells in the presence and absence of Fancl and proteosome inhibitors and conclude that Fancl promotes degradation of Tp53 via the proteosome. Their model is that Fancl acts in the ovary to prevent oocyte loss by ubiquitinating Tp53 and promoting its degradation via the proteosome. Further they propose that Dmrt1 negatively regulates fancl levels to promote oocyte loss and later testis differentiation. Although there are a few missing methods descriptions and some missing statistical analyses, overall, the data in the manuscript are clear, with appropriate numbers of individuals examined and statistical analysis, and are mostly consistent with the authors conclusions. However, the exciting conclusions are somewhat inaccessible, especially to the broader scientific readership, in the current draft. In particular in the abstract, introduction, and discussion sections.

**Response:** We appreciate the reviewer's efforts in reviewing the manuscript and providing constructive comments on the manuscript. We have performed additional experiments according to the suggestions, revised our manuscript thoroughly, and responded to the comments point by point in the current version of the manuscript (please see below).

Major:

1. The major concern is with the writing, which would benefit from major revision. I have listed a few examples by section below; however, more comprehensive revision to set up the questions and exciting conclusions more clearly would greatly strengthen the manuscript and make it accessible to nonexperts.

**Response:** We apologize for the writing problems. The manuscript has been carefully revised according to the suggestion raised by the reviewers and double-checked by us. Moreover, the modifications on the title (Line 1-2), abstract (Line 21-41), author summary (Line 42-55), introductions (Line 56-132), results (Line 133-272), discussions (Line 273-380) and materials and methods (Line 381-529) are made thoroughly in the revised manuscript.

2. Without measuring testosterone, estradiol in the various mutant contexts, it might be prudent to qualify some of the conclusions regarding “absence of androgen signaling” “absence of estradiol” etc. and instead stating that the specific genes are dispensable for ovary/testis determination, differentiation etc. This would allow for the possibility of other compensating enzymes or ways to make these steroid hormones. For example, it is fair to conclude that cyp17a1 is dispensable but is estradiol signaling completely eliminated or is it just reduced? Further, the authors and prior work indicate that some of the mutants have secondary sex trait deficits.

**Response:** We agree. Accordingly, we measured the plasma estradiol concentration in control female, control male, cyp17a1-/- fish, dmrt1-/- female, and cyp17a1-/-;dmrt1-/- fish using the Elisa kit (582251, Cayman Chemical Company, Ann Arbor, MI). The estradiol concentration in control male, cyp17a1-/- fish, and cyp17a1-/-;dmrt1-/- fish was significantly decreased compared with control female and dmrt1-/- female of the siblings (Figure 1O). In our previous work, the impaired male-typical secondary sex characters were displayed by the cyp17a1-/- zebrafish and common carp due to decreased testosterone concentration in plasma and testis (Ref. 1-4). The breeding tubercles on the pectoral fin, which is the male-typical secondary sex character, were not observed in the cyp17a1-/- fish, cyp17a1-/-;dmrt1-/- fish, indicating its decreased androgen levels due to cyp17a1-depletion.

However, we agree that the decreased concentration of estradiol could not be described as completely eliminated or absent in our previous version of the manuscript. This has been corrected in the current version of the manuscript (Line 1, 24, 54, 262, 277). For the remnant estradiol as we measured in the cyp17a1-/- fish (Figure 1O, lanes 4 and 5), we think that this may be caused by multiple reasons, including the brine shrimp as the food, baseline for the Elisa kit, or the plastic box system for fish rearing, etc.

**References:**

1. Zhai G et al. Androgen signaling regulates the transcription of anti-Müllerian hormone via synergy with SRY-Related Protein SOX9A. Science Bulletin. 2017;62 (3):197-203.

2. Zhai G et al. Characterization of sexual trait development in cyp17a1-deficient zebrafish. Endocrinology. 2018;159(10):3549-62.

3. Zhai G et al. Augmentation of progestin signaling rescues testis organization and spermatogenesis in zebrafish with the depletion of androgen signaling. eLife. 2022;28;11:e66118.

4. Zhai G et al. Successful production of an all-female common carp (Cyprinus carpio L.) population using cyp17a1-deficient neomale carps. Engineering. 2021;8,181-189.

3. Regarding the conclusions regarding Fancl and Tp53, because gonads and germ cells were not examined, it should be clearly stated that these are interactions and activities in 293 cells. Further, the conclusions with respect gonad development should be qualified to state that they are extrapolated from 293 to gonad. For example, “assuming similar regulatory relationships exist in the germline and gonads, these results provide novel insights into the regulatory network involved in Fancl functions for ovarian differentiation…”

**Response:** We agree. The conclusion has been revised according to this suggestion (Line 129-132, 318-322).

4. Regarding the interaction domain of Fancl, do truncations that eliminate multiple domains abolish or attenuate binding?

**Response:** We appreciate the suggestion. Accordingly, assessments of interaction with Tp53 were performed using additional truncated Fancl peptides. We provided evidence that both the WD repeat domain (WDR) and the C-terminal domain (CTD) of Fancl are essential domains required for its association with Tp53 (Line 245-247 and Figure 4D and E). This suggests that Fancl may bind to Tp53 in a manner dependent on multiple domains, i.e. the WDR and CTD domains.

5. For the western blots in Figure 3 and the supplemental figures, the abbreviation TCL should be defined in the legend.

**Response:** The abbreviation TCL represents total cell lysate. This has been corrected in the current version of the manuscript (Line 822, 831).

6. Appropriate allele designations need to be provided for all mutants analyzed.

**Response:** In fact, the loss-of-function alleles of cyp17a1 and dmrt1 in zebrafish generated by our group as previously described were used in this study (Ref. 1-4). Considering that all details of cyp17a1 and dmrt1 knockouts in zebrafish have been published in our previous papers, the mutant alleles were not detailed as shown in the figure in the current study. We agree that the relevant information is helpful to the readers, so the detailed information of the mutant alleles was provided in the Materials and Methods section (Line 388-390).

Since fancl and cyp17a1 are both located on chromosome 13, to obtain triple heterozygotes (cyp17a1+/-;dmrt1+/-;fancl+/-), CRISPR/Cas9-mediated cyp17a1 and fancl knockouts were performed in F2 embryos derived from mating dmrt1+/- females with dmrt1+/- males. The triple heterozygotes were then inbred to generate triple homozygotes (cyp17a1-/-;dmrt1-/-;fancl-/-). Therefore, in the current version of the manuscript, only the newly generated cyp17a1 and fancl null alleles in zebrafish by us are reported in this study (Line 398-403, Figure S2).

**References:**

1. Zhai G et al. Androgen signaling regulates the transcription of anti-Müllerian hormone via synergy with SRY-Related Protein SOX9A. Science Bulletin. 2017;62 (3):197-203.

2. Zhai G et al. Characterization of sexual trait development in cyp17a1-deficient zebrafish. Endocrinology. 2018;159(10):3549-62.

3. Zhai G et al. Augmentation of progestin signaling rescues testis organization and spermatogenesis in zebrafish with the depletion of androgen signaling. eLife. 2022;28;11:e66118.

4. Lin QH, et al. Distinct and cooperative roles of amh and dmrt1 in self-renewal and differentiation of male germ cells in zebrafish. Genetics. 2017;207(3):1007-22.

7. In Figure 1N and 2G, 4N “ratios” of various stages are reported. How were these ratios determined, from how many sections and individuals? Please clarify and describe this in the methods.

**Response:** Good suggestion. The staging system adopted for ovarian follicles was conducted based on the original definition of Selman et al as modified by the researchers (Ref. 1-6). For the accurate analysis of the oocyte stage designations, ovaries, which were dissected out from females of each genotype after anesthetization, were placed in a 60 mm culture dish containing 60% L-15 medium (Gibco, Carlsbad, CA, United States). The follicles of different stages were manually isolated and divided into five stages according to their size and vitellogenic stage: primary growth stage (~0.1 mm), previtellogenic stage (cortical alveolus, ~0.3 mm), early vitellogenic stage (~0.4 mm), midvitellogenic stage (~0.5 mm), and full grown but immature stage (~0.65 mm). The criteria we adopted for the staging of ovarian follicles were as follows:

We believe that this comprehensive analysis on the gross ovary dissected from the female fish is more accurate than the previous histological criteria. The references along with descriptions of our methods are provided in the Materials and Methods section (Line 418-427, Figure 1N).

**References:**

1. Selman K, et al. Stages of oocyte development in the zebrafish, Brachydanio rerio. J Morphol. 1993;218:203-224.

2. Wu K, et al. Disruption of dmrt1 rescues the all-male phenotype of the cyp19a1a mutant in zebrafish - a novel insight into the roles of aromatase/estrogens in gonadal differentiation and early folliculogenesis. Development. 2020;147(4).

3. Wang Y, et al. Developmental profiles of activin betaA, betaB, and follistatin expression in the zebrafish ovary: evidence for their differential roles during sexual maturation and ovulatory cycle. Biol Reprod. 2004;71(6):2056-64.

4. Pang Y, et al. Role of G protein-coupled estrogen receptor 1, GPER, in inhibition of oocyte maturation by endogenous estrogens in zebrafish. Dev Biol. 2010;342(2):194-206.

5. Chu L, et al. Targeted gene disruption in zebrafish reveals noncanonical functions of LH signaling in reproduction. Mol Endocrinol. 2014;28(11):1785-95.

6. Li J, et al. Zinc mediates the action of androgen in acting as a downstream effector of luteinizing hormone on oocyte maturation in zebrafish. Biol Reprod. 2019;100(2):468-78.

8. Figure 1R, relative to what? How was this normalized across samples? Please clarify and describe in the methods. Also, because comparisons are made between single and compound mutants, statistics should be included for Dmrt and double mutants as well.

**Response:** Thanks for pointing out this. The cyp17a1 heterozygote was bred with a dmrt1 heterozygote of the opposite sex to generate cyp17a1/dmrt1 double heterozygous fish, which were then inbred to generate an offspring population containing dmrt1-/- fish and cyp17a1-/-;dmrt1-/- fish. The females observed in cyp17a1+/+;dmrt1+/+ (control), cyp17a1+/+;dmrt1-/- (dmrt1-/-), and cyp17a1-/-;dmrt1-/- fish were adopted for the anatomical analysis and histological analysis and gene expression analysis. The control, dmrt1-/-, and cyp17a1-/-;dmrt1-/- fish at 17 and 23 dpf were adopted for gene expression analysis (Line 436-437). The fancl expression of the control female fish served as control. For each gene expression analysis in each sample, the housekeeping gene β-actin was used as endogenous reference gene, and the expression level of fancl was calculated as the fold change relative to β-actin. According to the suggestion, multiple comparisons of fancl expression in control, dmrt1-/- and cyp17a1-/-;dmrt1-/- fish at 17 and 23 dpf were performed using one-way ANOVA followed by Fisher's LSD test. This has been stated in the current version of the manuscript (Line 441-445).

The up-regulated fancl expression was also observed in dmrt1-/- fish at 17 and 23 dpf, however, it is not that significant as observed in the cyp17a1-/-;dmrt1-/- fish (Figure 2A and B). We interpret the highest up-regulation of fancl in cyp17a1-/-;dmrt1-/- fish to be the result of the compromised synergy of Dmrt1 and androgen signaling in inhibiting fancl transcription, as their synergistic effect in inhibiting the relative luciferase activity driven by fancl promoter (Figure 2).

9. Figure 1T-V, I understand that in situ on sections is challenging, but the image quality is not sufficient to reach a conclusion. In addition, quantitative conclusions are made based these experiments, but NBT:BCIP staining is not quantitative method since the researcher stops the reaction. Also, panel V is mislabeled as H on the figure.

**Response:** Thanks for pointing out this. The ISH using the antisense probe of fancl on the gonad cryosections of fish at 25 dpf has been re-conducted in triplets according to the suggestion (Figure 2). Based on the observations with an improved magnification and quality, the signals of the staining appeared in immature gonads of control fish were observed. This could be supported by the previous study concerning the expression and function of fancl in zebrafish (Ref. 1). It was also observed that fancl was abundantly expressed in the follicles of cyp17a1-/-;dmrt1-/- fish compared to the control female fish (Figure 2C and D). On the other hand, the cDNA fragment of 786 nt of fancl was used to synthesize the antisense probe as previously described (Ref. 1). Furthermore, no obvious signal of NBT:BCIP staining could be observed when hybridized with the sense probe of fancl (Figure S1, Line 172-180).

It should be noted that the glass slides containing the gonadal cryosections of fish of each genotype were placed in a 12 cm × 9 cm plastic box (please see below). This ensures that the manual manipulations are carried out under the same conditions, so that the reactions of the NBT:BCIP staining of the cryosections of immature gonads on each slide could be stopped simultaneously.

In fact, the intensity of in situ hybridization for comparative expression is common in the field, and the comparison of intensity between groups in this study was made based on previous descriptions (Ref. 2,3).

***cyp19a1a	amh***

However, we understand the concern of the reviewer and therefore the quantitative conclusions have been reported together with the qPCR results in the Results section (lines 168-172).

We apologize for the typographical error and the mislabeled panel V has been corrected to H. This has been updated in the current version of the manuscript (Figure 2).

**References:**

1. Rodriguez-Mari A, et al. Sex reversal in zebrafish fancl mutants is caused by Tp53-mediated germ cell apoptosis. PLoS Genet. 2010;6(7):e1001034.

2. Rodriguez-Mari A, et al. Characterization and expression pattern of zebrafish Anti-Mullerian hormone (Amh) relative to sox9a, sox9b, and cyp19a1a, during gonad development. Gene Expr Patterns. 2005;5(5):655-67.

3. Yan YL, et al. Gonadal soma controls ovarian follicle proliferation through Gsdf in zebrafish. Dev Dyn. 2017;246(11):925-45.

10. Figure 5E, statistics should be included for dmrt1 single mutants and triple mutants to distinguish between an effect due to loss of Dmrt1 alone as opposed to synergistic effects.

**Response:** Thanks for pointing out this. In the current version of the manuscript, the previous Figure 5 has been given up due to the following reasons: based on our observations in previous Figure 5, germ cells were mostly depleted in dmrt1-/- male fish at 90 dpf, and the presence of few germ cells still could be observed in a low proportion of males (previous Figure 5B and D, fewer green fluorescence than that of the control fish). The apoptotic germ cells were selected for statistical analysis and illustrated in previous Figure 5 only when its few Vasa-positive germ cells could be visualized in dmrt1-/- male fish and cyp17a1-/-;dmrt1-/-;fancl-/- fish; otherwise, it is difficult to distinguish the testis from other adipocyte tissues and impossible to detect the apoptosis of the germ cells.

The presence of the few Vasa-positive germ cells in dmrt1-/- male fish and cyp17a1-/-;dmrt1-/-;fancl-/- fish in our previous Figure 5 could be supported by the previous study of dmrt1-/- fish, which also possesses few to no germ cells (Ref. 1,2). However, we agree with reviewer #3 that few germ cells of the testis adopted for the immunofluorescence assay in the previous version of our manuscript may be not the objective reflection of the dysgenesis testis with lost germ cells in dmrt1-/- fish. To avoid confusing the readers, the previous Figure 5 has been given up (please see our response to minor comment 5 of reviewer #3).

**References:**

1. Webster KA, et al. Dmrt1 is necessary for male sexual development in zebrafish. Developmental Biology. 2017;422:33-46.

2. Lin QH, et al. Distinct and cooperative roles of amh and dmrt1 in self-renewal and differentiation of male germ cells in Zebrafish. Genetics. 2017;207(3):1007-22.

11. The models in Figure 6 are helpful, but a few genotypes are combined under the same scenarios based on the labels, but they are not really equivalent. For example, in scenario F, ,Dmrt1 and AR would still be present in Fancl loss of function, but taking away Dmrt1 doesn't suppress. It would help also to distinguish between fertile and sterile ovaries and testis in the various models because these are not functionally equivalent outcomes.

**Response:** We apologize for the presumptuous and ambiguous illustration of different knockout lines. To avoid reader confusion, the previous Figure 6 has been given up from the current version of the manuscript due to the above concerns, and this has not affected the conclusion of the manuscript.

12. In the methods under “transcriptome analysis” line 430, “At the indicated time points, total RNA from zebrafish gonads”. Please clarify if these were gonads or truncated bodies that contain gonads as described in the RNA extraction and qPCR section above.

**Response:** Thanks for pointing out this. Total RNA from the dissected ovaries of fish at 80 dpf was extracted using TRIzol reagent and performed transcriptome analysis. This has been clarified in the current version of the manuscript (Line 447).

Minor:

14. Supporting figure S1 and S3 are nice but not necessary.

**Response:** We agree. Accordingly, the previous Figure S1 and S3 have been deleted in the current version of the manuscript.

Writing:

Abstract:

1. “The laboratory strains of zebrafish lack a typical sex chromosome, and the gonadal differentiation genes downstream of a sex determination gene that regulates the animals develop as males or females, though conserved among vertebrates, is still unknown yet in zebrafish.” This is a complex sentence with many ideas that can be separated into simpler sentences.

**Response:** This sentence has been re-written as “The regulatory mechanism of gonadal sex differentiation, which is complex and regulated by multiple factors, remains poorly understood in teleosts.” (Line 22-23).

2. From the abstract, “exclusive gonadal sex shift from the testes to the ovaries was caused by depletion of doublesex and mab-3 related transcription factor 1 (dmrt1) in cyp17a1-/- zebrafish.” This is potentially misleading as written as it might imply the double mutants make a testis that then becomes an ovary. And later “cyp17a1-/-;dmrt1-/- zebrafish, comparative gonadal transcriptome analysis revealed that the phenotypes shifting from testes to ovaries correlated with up-regulated gonadal fancl.”

**Response:** This sentence has been re-written as “the female-biased sex ratio phenotypes were positively correlated with upregulated levels of Fanconi anemia complementation group L (fancl) in the gonads of doublesex and mab-3 related transcription factor 1 (dmrt1)-/- and cyp17a1-/-;dmrt1-/- fish.” (Line 26-31).

3. “Mechanistically, the degradation of TP53 with the activation of its K48-linked polyubiquitination within its DNA-binding domain (DBD), mediated via the ubiquitin ligase Fancl, has been demonstrated.” This sentence is unclear.

**Response:** Thanks for pointing out this. This has been re-written as “The interaction between Fancl and Tp53 was observed via the WD repeat domain (WDR) and C-terminal domain (CTD) of Fancl and the DNA binding domain (DBD) of Tp53, leading to the K48-linked polyubiquitination degradation of Tp53 activated by the ubiquitin ligase, Fancl.” (Line 34-37).

4. “Taken together, our results revealed that Dmrt1 signaling determines testis fate for gonadal differentiation even in the absence of androgen synthesis in cyp17a1-/- fish, which antagonizes Fancl/TP53 signaling during the critical stage..” This is a complex sentence and seems inconsistent with the previous sentence states that the triple mutants male even in the absence of Dmrt1 and Cyp17a1.

**Response:** Thanks for pointing out this. This has been re-written as “Our results show that testis fate in cyp17a1-/- fish is determined by Dmrt1, which is thought to stabilize Tp53 by inhibiting fancl transcription during the critical stage of sexual fate determination in zebrafish.” (Line 37-40).

Author summary:

1. “Without the presence of testosterone and estradiol in cyp17a1-deficient zebrafish, Dmrt1 signaling sufficiently promotes the all-testis differentiation via activating germ cell apoptosis mediated through TP53 by suppressing the expression of fancl.”

**Response:** This has been re-written as “By analyzing a series of mutant zebrafish lines, we show here that Dmrt1 sufficiently promotes all-testis differentiation in cyp17a1-deficient zebrafish albeit the compromised androgen and estrogen synthesis. In addition, we observed that zebrafish Dmrt1 and androgen signaling probably stabilize Tp53 by inhibiting the transcription of a ubiquitin ligase, Fancl.” in the current version of the manuscript (Line 48-53).

Introduction:

1. “Besides, unlike the female biased ratio phenotype and impaired spermatogenesis seen in ar-/- zebrafish [18], all-testis differentiation and normal spermatogenesis have been achieved in our cyp17a1-/-;ar-/- zebrafish.” This is unclear as written and seems inconsistent with the statements later in the paragraph thar ar and androgen is dispensable.

**Response:** Thanks for pointing out this and this has been deleted accordingly. Currently, for androgen receptor, only the synergistic effect of Dmrt1 and androgen signaling in determining testis fate via inhibiting fancl expression have been retained and discussed in the current version of the manuscript (Line 310-314).

2. “Moreover, the interaction between Fancl and TP53 led to the discovery of the activation of K48- linked ubiquitination of TP53, which contributes to the survival of germ cells in zebrafish during gonadal differentiation.” This is misleading as written because the interactions and modifications were demonstrated for somatic cells, 293, and not for germ cells. Further it is unclear as written if the authors are referring to this work or other work.

**Response:** This has been corrected in the current version of the manuscript (Line 129-132).

3. “Dmrt1 can repress fancl expression, disrupt Fancl/TP53 interaction, promote apoptosis in germ cells during the critical gonadal differentiation window period, and determine all-testis differentiation, without the antagonistic effect of estradiol signaling in cyp17a1-/- zebrafish.” This sentence mixes multiple contexts and is not fully consistent with the data shown.

**Response:** This has been corrected in the current version of the manuscript (Line 127-128).

Discussion:

1. The authors report results including new results and refer back to the earlier figures 1 and 2. This is not typical of a discussion section which is typically a synthesis of results. Usually, the only figures referred to are new model or summary figures.

**Response:** We apologize for the inaccurate format of the discussion section. This has been corrected in the current version of the manuscript (please see discussion section).

2. “However, when the nuclear progestin receptor (npgr) was deleted in cyp17a1-/-;ar-/- fish, the phenotype of all-testis differentiation was sustained even with impaired spermatogenesis in cyp17a1-/-;ar-/-;npgr-/- zebrafish.” “even with” should be “albeit with”

**Response:** This has been corrected in the current version of the manuscript (Line 50, 262, 277, 289).

3. “This indicates that Dmrt1 signaling can still cancel the testis fate for gonadal differentiation, under testosterone and estradiol absence in cyp17a1-/- zebrafish.” This is inconsistent with the data shown and is potentially confusing phrasing. The data show Dmrt1 is required for testis fate not to cancel it.

**Response:** We apologize for the confusing description here. This has been corrected in the current version of the manuscript (Line 288-290).

4. “Therefore, it is reasonable to speculate that the synergistic effects on testis fate determination could be ensured by both discrete Dmrt1 and androgen signaling in our cyp17a1-/-;dmrt1-/- zebrafish”. This sentence is unclear as written.

**Response:** This has been corrected in the current version of the manuscript (Line 305-314).

5. “It is in lines with our previous view that Dmrt1 is required for the maintenance, self-renewal, and differentiation of male germ cells, and implying that in cyp17a1-/-;dmrt1-/- fish, the additional depletion of fancl resulted in sex reversal from ovaries to testes, which was compromised due to dmrt1-deficiency, which in turns, caused dysregulated male germ cells development afterwards.” This is a complex sentence with many ideas that can be separated into simpler sentences.

**Response:** This has been corrected in the current version of the manuscript (Line 323-327).

6. “Depletion of tp53 (IHB136, China Zebrafish Resource Center) did not lead to a female-biased zebrafish population and did not reverse all-testis differentiation in cyp17a1-/- zebrafish (S5 Fig), suggesting that TP53 signaling might only provide complementary, sustainable, but not decisive, roles for ovarian differentiation in zebrafish.” This is unclear. Do the authors mean to say that it is only a factor in the context of ovarian failure and not normal sex determination and differentiation.

**Response:** Thanks for pointing out this. This sentence has been corrected in the current version of the manuscript (Line 340-347).

7. “Our results further indicate that Dmrt1 is a key determining factor for testis differentiation in certain fish even in the absence of testosterone nor estradiol, partially via the suppression of fancl expression, which leads to elevated TP53-induced germ cell apoptosis in zebrafish (Fig 6).” This may be a bit confusing. It might help to add something about when Tp53-induced death of oocytes occurs during ovarian failure or in the bipotential gonad.

**Response:** Thanks for pointing out this. As the previous Figure 6 has been given up according to the reviewer’s suggestion (please see our response to major comment 11), this sentence has been deleted in the current version of the manuscript.

8. “S2 Fig. Every single domain mutation of Fancl did not affect its association with TP53.”

**Response:** This caption has been changed to “S3 Fig. The single domain mutation of Fancl did not affect its association with Tp53.”.

## *Response to Reviewer 2:*

**Reviewer #2:**

Previous work from this lab showed that, remarkably, cyp17a1 mutants, which cannot produce either the male-promoting hormone testosterone or 11KT, or the female-promoting hormone 17ß-estradiol, all develop as fertile males. Iin this present study they extend these findings by analyzing the phenotype of cyp17a1;dmrt1 double mutants. dmrt1 is a highly conserved regulator of male development and all dmrt1 zebrafish mutants are fertile females. The authors found that dmrt1;cyp17a1 double mutants develop as females. However, these females are presumably sterile as the oocytes they produce never progress past stage IB (primary growth stage). They further show that oocyte development can be rescued in the double mutants by estradiol treatment. By comparing the transcriptomes of normal and double mutant ovaries they identify many genes that are differentially regulation. One in particular is fancl, which was previously shown to be required for female development by suppression of Tp53-mediated germ cell apoptosis. Using proteomic analysis, they demonstrate that Fancl directly interacts with the DNA binding domain of Tp53, and that it can promote polyubiquitinate on K48. They further show that polyubiquitinate leads to the degradation of Tp53, thus providing a mechanistic model for the role of Fancl in ovary development. Finally, they show that removing fancl function in cyp17a1;dmrt1 mutants, which was not a trivial task given that fancl and cyp17a1 are on the same chromosome, lead to an all sterile male phenotype.

Two main conclusions can be drawn from this data: 1) In the absence of dmrt1 and androgen production, estrogen production is not required for ovary differentiation in zebrafish, but is required for oocytes to progress past stage IB. 2) Suppression of fancl expression by Dmrt1 is necessary for male/testis development. The data in the paper are clear and well presented, and the results will be of interest to a wide audience. The comments/suggestion below are mostly minor and intended to increase the clarity of the paper.

**Response:** We appreciate the reviewer’s efforts in reviewing the manuscript and providing the constructive comments on the manuscript. We have revised our manuscript according to the suggestions point by point in the current version of the manuscript (please see below).

L47: …Dmrt1 signaling… (it is not a signal.) Better to say …Dmrt1 transcriptional regulation…

**Response:** Thank you for pointing out this. This has been corrected in the current version of the manuscript (Line 45-46).

L68: “Cyp17A1 is a key enzyme involved in testosterone synthesis in animals.” It would be good to point out here that Cyp17a1 is required for both testosterone and estrogen production, as this is important to understanding the result presented here.

**Response:** Thank you for pointing out this. This has been corrected in the current version of the manuscript (Line 76-78).

L102: For completeness in referencing primary publications, please add Dranow et al., 2016, which also analyzed cyp19a1a mutants.

**Response:** The suggested reference has been added (Line 73).

L104: For completeness in referencing primary publications, please add Romano et al., 2020, which also analyzed the cyp19a1a;dmrt1 double mutants.

**Response:** The suggested reference has been added (Line 76).

L101-111: A more appropriate reference for this statement is Tzung et al., 2015.

**Response:** The suggested reference has been added (Line 96).

L112: For completeness in referencing primary publications, please add Siegfried and Nusslein-Volhard, 2008, which also analyzed sex determination in dnd morphants.

**Response:** The suggested reference has been added (Line 98).

L117: For completeness in referencing primary publications, please add Tzung et al., 2015.

**Response:** The suggested reference has been added (Line 102).

L117: It would be more accurate to say “…specific levels of signals derived from oocytes…” as it is oocytes, not pre-meiotic germ cells, that are required for female development (see also Rodriguez-Mari et al., 2010).

**Response:** We agree. This sentence has been corrected according to this suggestion (Line 102-104).

L132: More accurate to say: “…surviving oocytes…”

**Response:** Thank you for pointing out this. This has been corrected in the current version of the manuscript (Line 117).

L141: “mysterious” is not a scientific term and should be avoided.

**Response:** Thank you for pointing out this. This has been deleted in the current version of the manuscript (Line 124).

L165 & L280: For completeness in referencing primary publications, please add Webster et al., 2017, as they also showed dmrt1-/- fish are primarily female and the few males are sterile.

**Response:** The suggested reference has been added (Line 224).

Fig 1: This labels for the various oocyte stages appear accurate based on histological criteria, but the scale bar in Fig 1G does not appear to be accurate. Based on this bar, the FG oocytes are ~80-90 µm in diameter, which is the size of primary growth stage oocytes (Selman et al. 1993). FG oocytes should be 730-750 µm. Perhaps the bar is actually 50 µm. Please confirm all scale bars are accurate.

**Response:** We apologize for the inaccurate scale bars provided in the previous Figures due to the setup error we made. The scale bars have been corrected in the current version of the Figures and double-checked by us (Figure 1, 2, 3 and 6). The oocytes at 50 and 90 dpf are shown with with a scale bar of 50 and 300 μm, respectively. Normal testes are shown with a scale bar of 25 μm, and the hypoplastic testes are shown with a scale bar of 10 μm (Line 410-412).

Fig 1: Please consider changing the oocyte stage designations (i.e. PG, EV, FG) to that of Selman et al., (1993) as it allows you to define the oocyte stages more precisely. For example, it is stated that cyp17a1;dmrt1 double mutant ovaries contain only pre-vitellogenic oocytes. However, pre-vitellogenic oocytes encompass pre-follicle (Stage 1A), follicle stage/primary growth stage (Stage IB), and cortical alveolus stage (Stage II) oocytes. Based on Fig 1L, cyp17a1;dmrt1 ovaries do not contain any oocytes that have progressed passed Stage IB. Thus, use of the Selman et al., staging allows a more precise description of the phenotype.

**Response:** We appreciate this suggestion. The staging system adopted for ovarian follicles was conducted based on the original definition of Selman et al as modified by the researchers (Ref. 1-6). For the accurate analysis of the oocyte stage designations, ovaries, which were dissected out from females of each genotype after anesthetization, were placed in a 60 mm culture dish containing 60% L-15 medium (Gibco, Carlsbad, CA, United States). The follicles of different stages were manually isolated and divided into five stages according to their size and vitellogenic stage: primary growth stage (~0.1 mm), previtellogenic stage (cortical alveolus, ~0.3 mm), early vitellogenic stage (~0.4 mm), midvitellogenic stage (~0.5 mm), and full grown but immature stage (~0.65 mm). The criteria we adopted for the staging of ovarian follicles were as follows:

We believe that this comprehensive analysis on the gross ovary dissected from the female fish is more accurate than the previous histological criteria. The references along with the descriptions of our methods have been provided in the Materials and Methods section (Line 418-427, Figure 1N).

**References:**

1. Selman K, et al. Stages of oocyte development in the zebrafish, Brachydanio rerio. J Morphol. 1993;218:203-224.

2. Wu K, et al. Disruption of dmrt1 rescues the all-male phenotype of the cyp19a1a mutant in zebrafish - a novel insight into the roles of aromatase/estrogens in gonadal differentiation and early folliculogenesis. Development. 2020;147(4).

3. Wang Y, et al. Developmental profiles of activin betaA, betaB, and follistatin expression in the zebrafish ovary: evidence for their differential roles during sexual maturation and ovulatory cycle. Biol Reprod. 2004;71(6):2056-64.

4. Pang Y, et al. Role of G protein-coupled estrogen receptor 1, GPER, in inhibition of oocyte maturation by endogenous estrogens in zebrafish. Dev Biol. 2010;342(2):194-206.

5. Chu L, et al. Targeted gene disruption in zebrafish reveals noncanonical functions of LH signaling in reproduction. Mol Endocrinol. 2014;28(11):1785-95.

6. Li J, et al. Zinc mediates the action of androgen in acting as a downstream effector of luteinizing hormone on oocyte maturation in zebrafish. Biol Reprod. 2019;100(2):468-78.

L170: “These results suggest…” This statement needs more context for the general audience to understand. Please explain the logic behind the conclusion that estradiol signaling is important for maturation but not differentiation.

**Response:** Thanks for pointing out this. This has been corrected as in the current version of the manuscript (Line 147-155).

Fig. 1T-H: How many independent gonads were examined and are the images in T-H representative? Please give n’s for each genotype. This is mainly a concern for the control gonads, because some may have already begun to transition to testes and may therefore have downregulated fancl. The image in U is of poor quality due to the many salt crystals that formed over the tissue. This panel should be replaced with a higher quality image. Please add labels for oocytes, if they are present (e.g. it looks like a stage IB oocyte is present in H).

**Response:** Thanks for pointing out this. The ISH using the probe of fancl on the gonad cryosections of fish at 25 dpf has been re-conducted in triplets according to the suggestion (Figure 2). Based on the observations with an improved magnification and quality, the signals of the staining appeared in immature gonads of control fish were observed.

The new panels C and D were representative, as they were run in triplicate. This could be supported by the previous study concerning the expression and function of fancl in zebrafish (Ref. 1). It was also observed that fancl was abundantly expressed in the follicles of cyp17a1-/-;dmrt1-/- fish compared to the control female fish (Figure 2C and D). On the other hand, the cDNA fragment of 786 nt containing the PHD domain of fancl was used to synthesize the antisense probe as previously described (Ref. 1). Furthermore, no obvious signal of NBT:BCIP staining could be observed when hybridized with the sense probe of fancl (Figure S1, Line 172-180).

We agree that the presumptive ovary and presumptive testis begin to undergo distinct developmental processes from 25 to 30 dpf. Thus, the gonads which only exhibit typical presumptive ovary structure was selected for the analysis. The immature oocytes have been labelled with arrows according to the suggestion (Figure 2C and D).

**References:**

1. Rodriguez-Mari A, et al. Sex reversal in zebrafish fancl mutants is caused by Tp53-mediated germ cell apoptosis. PLoS Genet. 2010;6(7):e1001034.

L325: “Therefore it is…” The point being made by the sentence is not clear to this reviewer.

**Response:** Thank you for pointing out this. This has been corrected in the current version of the manuscript (Line 294-304).

L334: cyp17a1-/- should be dmrt1-/-

**Response:** Thank you for pointing out this. This has been corrected in the current version of the manuscript (Line 309).

## *Response to Reviewer 3:*

**Reviewer #3:**

The gene network that directs gonad to develop into ovary or testis is far from clear in zebrafish as well as in other vertebrates. In this study, the authors demonstrate that Dmrt1 determines the testis fate of gonads possibly via antagonizing Fancl/TP53 signaling even in the absence of androgen synthesis in cyp17a1-/- fish. Firstly, the authors analyzed the gonadal phenotypes of cyp17a1-/-, dmrt1-/- single mutants and cyp17a1-/-;dmrt1-/- double mutants in zebrafish. They showed that dmrt1 deletion reversed the all-testis phenotype of cyp17a1-/- zebrafish into all-ovary. Next, by comparing gene expression in the gonads of cyp17a1-/-;dmrt1-/- fish and control fish and rescue experiments by fancl (FA signaling gene) mutation, they showed that the up-regulation of fancl was responsible for the all-ovary development of cyp17a1-/-;dmrt1-/- zebrafish. Finally, they proved that fancl was involved in the regulation of germ cell apoptosis by mediating the degradation of the apoptotic factor TP53 via activating K48-linked polyubiquitination in the DNA binding domain. This study provides additional insights for zebrafish gonadal differentiation. The study is innovative, and the experiments are designed properly. However, several concerns should be addressed before publication.

**Response:** We appreciate the reviewer’s efforts in reviewing the manuscript and providing the constructive comments on the manuscript. We have revised our manuscript according to the suggestions point by point in the current version of the manuscript (please see below).

**Major:**

1. The most attractive point of this manuscript is that the authors found Dmrt1 can antagonize Fancl/TP53 signaling to regulate gonad differentiation in cyp17a1-/- zebrafish with neither estrogen nor androgen. Considering that the expression of Fancl in developing germ cells and its function in TP53-mediated germ cell apoptosis and gonadal differentiation have been reported in zebrafish previously (Rodríguez-Marí et al., 2010), the authors need to focus their research on the relationship between Dmrt1 and Fancl, rather than studying how Fancl affects TP53. Is it possible to prove that Dmrt1 can directly suppress fancl expression and Fancl can mediate the degradation of Dmrt1 via polyubiquitination in vitro?

**Response:** Thank you for pointing this out. We agree that the relationship between Dmrt1 and Fancl should be focused and better explained to support our statement. Unfortunately, there are no commercialized antibodies available against Dmrt1 and Fancl. Therefore, it is difficult for us to investigate the potential endogenous interaction between Dmrt1 and Fancl molecule or fancl promoter region. Accordingly, the promoter was cloned into pGL3-basic plasmid, which was co-transfected into the HEK293T cells with the pcDNA3.1-Dmrt1 and DHT/pcDNA3.1-Ar. Both Dmrt1 and DHT/Ar inhibited the relative luciferase activity driven by the fancl promoter, and their combination treatment resulted in the greatest inhibitory effect in vitro (Figure 2H).

On the other hand, in our analyses, compared with those in control females, the elevated expression of fancl in presumptive ovaries of cyp17a-/-;dmrt1-/- fish at 17, 23 and 25 dpf was positively correlated with its female-biased sex ratio. Actually, a moderated up-regulation of fancl in dmrt1-/- fish at 17-23 (Figure 2A and B) and 80 dpf (please see below, indicated with red box) was also observed by us. However, it is not that significant as observed in the cyp17a1-/-;dmrt1-/- fish (Figure 2A and B). We interpret the highest up-regulation of fancl in cyp17a1-/-;dmrt1-/- fish to be the result of the compromised synergy of Dmrt1 and androgen signaling in inhibiting fancl transcription, as their synergistic effect in inhibiting the relative luciferase activity driven by fancl promoter (Figure 2H, Line 198-201).

The degradation of Dmrt1 mediated by Fancl was not detected by us when they were transfected into HEK293T cells. Meanwhile, we observed that the dmrt1-/-;fancl-/- fish also has a hypoplastic testis with lost germ cells, similar to that of the dmrt1-/- fish, compared to the control fish (Figure 3). The severely hypoplastic testes of dmrt1-/- fish, dmrt1-/-;fancl-/- fish and cyp17a1-/-;dmrt1-/-;fancl-/- fish is in line with our previous view that Dmrt1 is required for the maintenance of male germ cells. This could be interpreted as additional fancl depletion further promoting testis differentiation in dmrt1-/- fish (i.e., the dmrt1-/-;fancl-/- fish) and cyp17a1-/-;dmrt1-/- fish (i.e., the cyp17a1-/-;dmrt1-/-;fancl-/- fish), but their male germ cells development was dysregulated due to dmrt1-deficiency (Line 328-332). There results suggest that the additional depletion of fancl switches the fate of dmrt1-/- fish gonads to testis (in dmrt1-/-;fancl-/- fish) and strengthen the notion that fancl is the downstream regulator of dmrt1 in determining testis fate (Line 228-232).

2. The transcriptome data displayed in the results section of this manuscript is not persuasive enough to convince readers that fancl up-regulation is the main reason for the all-ovary phenotype of cyp17a1-/-;dmrt1-/- zebrafish. Gonads should be sampled at early stage of gonadal differentiation rather than at 80 dpf.

**Response:** We agree that gonads should be sampled at an early stage of gonadal differentiation. However, without proper marker-labelled gonadal tissue at that time, it is difficult to dissect the immature, especially most of the samples were severely hypoplastic gonads of small size, to harvest sufficient amount of RNA for transcriptomic analyses. Our transcriptomic and qPCR data for gene expression analyses in fish at 17 and 23 dpf could only be performed using the truncated zebrafish bodies, which contained the gonads and parts of other tissues. Fortunately, all the genes we were interested in are gonadal or germ cell specific.

To rule out the possibility that our quantification analyses was caused by the RNA sample of the control gonads which may contaminated from other tissues or different stage, we also analyzed the expression patterns of fancl by in situ hybridization in presumptive ovaries from control fish and cyp17a1-/-;dmrt1-/- fish at 25 dpf. The moderate expression of fancl in control fish gonads, but up-regulated expression in cyp17a1-/-;dmrt1-/- fish gonads were observed (Figure 2). On the other hand, the cDNA fragment of 786 nt containing the PHD domain of fancl was used to synthesize the antisense probe as previously described (Ref. 1). No obvious signal of NBT:BCIP staining with the sense probe of fancl has been tested, indicating the transcripts of fancl analyzed were expressed in the gonad tissue samples (Figure S1, Line 172-180).

**References:**

1. Rodriguez-Mari A, et al. Sex reversal in zebrafish fancl mutants is caused by Tp53-mediated germ cell apoptosis. PLoS Genet. 2010;6(7):e1001034.

The up-regulation of fancl might be the consequence rather than the reason of sex reversal. Also, to find out the key gene that reversed the all-testis phenotype of cyp17a1-/- fish into all-ovary after dmrt1 deletion, gonadal gene expression should be compared between cyp17a1-/-;dmrt1-/- fish and cyp17a1-/- fish and between cyp17a1-/-;dmrt1-/- fish and dmrt1-/- fish, rather than between cyp17a1-/-;dmrt1-/- fish and wild type female fish.

**Response:** To identify the factor potentially responsible for male fate determination in cyp17a1-/- fish, dmrt1-/- fish were obtained from the laboratory of Dr. Jie Mei (Ref. 1). The cyp17a1-/-; dmrt1-/- fish were generated by crossing dmrt1-/- and cyp17a1-/- lines. It was then found that the different phenotypes of pro-ovarian differentiation between dmrt-/- fish (rate of ovarian differentiation is about 75%) and cyp17a1-/-;dmrt1-/- fish (all-ovarian differentiation). To identify the key factor responsible for the switch from all-male to all-female upon additional depletion of dmrt1 in cyp17a1-/- fish, several potential or known ovarian differentiation-related genes were selected based on the recently reported landscape of differentiated biological processes involved in initiation (Ref. 2). Fancl was selected as one of the candidate genes for transcriptional analysis. It was then found that the up-regulated expression levels of fancl were corresponding to the ratios of ovarian differentiation in the dmrt1-/- fish and cyp17a1-/-; dmrt1-/- fish. Due to the severely hypoplastic gonads in cyp17a1-/-;dmrt1-/- fish, great efforts were made to harvest a sufficient amount of RNA samples at 80 dpf stage for transcriptomic analyses. Fortunately, fancl was found to be one of the few significantly up-regulated transcripts in the gonads of cyp17a1-/-; dmrt1-/- fish compared to control female fish. Combined with the expression characteristics of fancl at 17, 23, 25 and 80 dpf stages and the fancl:luciferase assay analysis, it is convincing to link the sex determination phenotypes observed in cyp17a1-/-, dmrt1-/- and cyp17a1-/-; dmrt1-/- fish to the induction of fancl expression. There results suggest that the additional depletion of fancl switches the fate of dmrt1-/- fish gonads to testis (in dmrt1-/-;fancl-/- fish) and strengthen the notion that fancl is the downstream regulator of dmrt1 in determining testis fate (Lines 228-232).

Without labelled gonadal cells for tracking, it is difficult to technically collect sufficient amounts of RNA from mutant fish at 25 dpf for transcriptomic analyses. If the harvest time is set at 80 dpf, all gonads develop into testes in cyp17a-/- fish, whereas all of them develop into ovaries in cyp17a1-/-;dmrt1-/- fish. The testis RNA sample from cyp17a1-/- fish might not be suitable as a control compared to the ovarian RNA sample from cyp17a1-/-;dmrt1-/- fish. Therefore, we chose the ovarian RNA samples from cyp17a1-/-;dmrt1-/- fish and control female fish for the transcriptomic comparison.

**References:**

1. Lin QH, et al. Distinct and cooperative roles of amh and dmrt1 in self-renewal and differentiation of male germ cells in zebrafish. Genetics. 2017;207(3):1007-22.

2. Ye D, et al. A landscape of differentiated biological processes involved in the initiation of sex differentiation in zebrafish. Water Biology and Security. 2022;1:100059.

3. It is widely accepted that estrogen but not androgens plays essential role in fish sex determination and differentiation. Blocking estrogen synthesis led to female to male sex reversal and estrogen administration led to male to female sex reversal in many fish species, including zebrafish. There has been no report showing that blocking androgen synthesis changes sex in fish. The all-testis phenotype of cyp17a1-/- and cyp17a1-/-;ar-/- zebrafish in this study should be explained by estrogen deficiency. It has nothing to do with androgen or progestin augmentation. The authors should change their description in the Title, Abstract (line 24-27) and Discussion (298-303) section of this manuscript to avoid misleading the readers.

**Response:** For this comment excluding androgen in testis fate determination, we would like to present as follows:

Both cyp17a1-/- and cyp19a1a-/- fish develop into males due to estrogen deficiency, and the depletion of cyp19a1a in zebrafish and its developmental consequences have been reported by different groups (Ref. 1,2). However, our cyp17a1-/- zebrafish still provides us with a unique model to uncover several novel findings that were never noticed in the cyp19a1a-deficient fish.

It has been reported that the ratio of ovarian differentiation in cyp19a1a-/-;dmrt1-/- zebrafish decreases progressively from 60 to 100 dpf, with female ratios of 83%, 43% and 13% at 60, 75 and 100 dpf, respectively (Ref. 3). In contrast, the ratio of ovarian appearance in cyp17a1-/-;dmrt1-/- fish at 90 dpf was 100% (Figure 1M). On the other hand, the ratio of ovarian differentiation in dmrt1-/- zebrafish is approximately 70-80% (Ref. 4,5) (Figure 1M). All previous results have been concerned with the inhibitory role of estrogen signaling on Dmrt1 for ovarian differentiation (Ref 6,7). However, using the cyp17a1-/-; dmrt1-/- fish, our data reveal a minor but not negligible role for androgen signaling in fish sex determination and differentiation. Using previously published profiles, we compared endogenous steroid levels between cyp17a1-/- and cyp19a1a-/- fish. Testosterone was impaired and accumulated in cyp17a1-/- and cyp19a1a-/- fish, respectively (Ref. 8-10). Compared to cyp19a1a-/-;dmrt1-/- fish, it is reasonable to speculate that the higher ratio of ovarian differentiation in cyp17a1-/-;dmrt1-/- fish is due to impaired androgen synthesis. In other words, the synergistic effects of simultaneous depletion of Dmrt1 and androgen synthesis may exist in promoting gonadal embarkation on ovarian differentiation. This is in agreement with the phenotype reported in ar-/- zebrafish, in which the differentiation of the ovary is biased, but not 100%. This suggests that androgen signaling may indeed antagonize ovary differentiation in zebrafish (Ref. 9,11).

In our analyses, the increased expression levels of fancl in presumptive ovaries of cyp17a-/-;dmrt1-/- fish at 17, 23 and 25 dpf compared to control females were positively correlated with their female biased sex ratios (Figure 2). Indeed, we also observed a moderate up-regulation of fancl in dmrt1-/- fish at 17-23 and 80 dpf (please see our response to major comment 1). We interpret this phenotype as the result of the synergistic effect of Dmrt1 and androgen signaling in inhibiting fancl transcription, as their synergistic effect in inhibiting relative luciferase activity driven by the fancl promoter (Figure 2H).

All of the above and constructive suggestions have been carefully considered. They have been incorporated into our current version of the manuscript.

**References:**

1. Yin Y, et al. Targeted disruption of aromatase reveals dual functions of cyp19a1a during sex differentiation in zebrafish. Endocrinology. 2017;158(9):3030-41.

2. Lau ES, et al. Knockout of Zebrafish Ovarian Aromatase Gene (cyp19a1a) by TALEN and CRISPR/Cas9 Leads to All-male Offspring Due to Failed Ovarian Differentiation. Sci Rep. 2016;6:37357.

3. Wu K, et al. Disruption of dmrt1 rescues the all-male phenotype of the cyp19a1a mutant in zebrafish - a novel insight into the roles of aromatase/estrogens in gonadal differentiation and early folliculogenesis. Development. 2020;147(4).

4. Webster KA, et al. Dmrt1 is necessary for male sexual development in zebrafish. Dev Biol. 2017;422(1):33-46.

5. Lin Q, et al. Distinct and Cooperative Roles of amh and dmrt1 in Self-Renewal and Differentiation of Male Germ Cells in Zebrafish. Genetics. 2017;207(3):1007-22.

6. Wu K, et al. Disruption of dmrt1 rescues the all-male phenotype of the cyp19a1a mutant in zebrafish - a novel insight into the roles of aromatase/estrogens in gonadal differentiation and early folliculogenesis. Development. 2020;147(4).

7. Romano S, et al. Loss of dmrt1 restores zebrafish female fates in the absence of cyp19a1a but not rbpms2a/b. Development. 2020;147(18).

8. Zhai G, et al. Characterization of sexual trait development in cyp17a1-deficient zebrafish. Endocrinology. 2018;159(10):3549-62.

9. Yu G, et al. Zebrafish androgen receptor is required for spermatogenesis and maintenance of ovarian function. Oncotarget. 2018;9(36):24320-34.

10. Yin Y, et al. Targeted Disruption of Aromatase Reveals Dual Functions of cyp19a1a During Sex Differentiation in Zebrafish. Endocrinology. 2017;158(9):3030-41.

11. Crowder CM, et al. Nuclear androgen receptor regulates testes organization and oocyte maturation in zebrafish. Endocrinology. 2018;159(2):980-93.

4. Expression of fancl is upregulated in the dmrt1-/- fish. How about the gonadal development in the dmrt1-/-;fancl-/- fish? The authors should provide these data.

**Response:** Thank you for your suggestion. Accordingly, the dmrt1-/-;fancl-/- fish were used for anatomical and histological analysis. The dmrt1-/-;fancl-/- fish also have a hypoplastic testis with germ cell loss similar to that of the dmrt1-/- fish (Figure 3). There results suggest that the additional depletion of fancl switches the fate of dmrt1-/- fish gonads to testis (in dmrt1-/-;fancl-/- fish) and strengthen the notion that fancl is the downstream regulator of dmrt1 in determining testis fate (Line 228-232)

**Minor:**

1. Line 183-185, “The top enriched pathways were related to FA, steroid hormone biosynthesis, apoptosis, Notch signaling, Toll-like receptor signaling and mTOR signaling”. The order of the enriched pathway should be written according to either the gene numbers or the significance of pathways enriched. Also, the criteria for the identification of fancl as the key regulators need to be clarified.

**Response:** Thanks for pointing out this. The order of the enriched pathway was described according to the significance of pathways enriched (Line 187-189). The relevant descriptions in identifying fancl has been provided in the current version of the manuscript (Line 157-195).

2. Line 260-269, “However, the introduction ...... with a 37 bp deletion in the ninth exon”. This part should be move to the Materials and Methods section.

**Response:** This has been corrected in the current version of the manuscript (Line 398-403).

3. This MS focus on sex differentiation of zebrafish mutants. The authors should show the phenotype at different stages of gonadal development in these mutants. Not just only at adult stage.

**Response:** We agree with this suggestion. The histological analysis of the gonads of control fish, cyp17a1-/- fish, dmrt1-/- fish and cyp17a1-/-;dmrt1-/- fish at 50 dpf were provided. Analysis of sex ratios in fish at 50 dpf showed that the cyp17a1-/- fish were all males, whereas all of the cyp17a1-/-;dmrt1-/- fish developed into females (Figure 1R, U and V). Compared with the control and dmrt1-/- female fish, PG follicles of the early folliculogenesis occurred normally in cyp17a1-/-;dmrt1-/- fish (Figure 1P, S and U) (Line 149-155).

4. In Figure 1Q, please show the expression of fancl in dmrt1-/- fish at 80 dpf? In Figure 1S, why fancl was not upregulated in the dmrt1-/- fish compared with control? In Figure 1T, no sense probe was shown as control. The signal was not specific in germ cell, but with a universal expression pattern. Why? It has been reported that fancl is specifically expressed in the germ cells (Rodríguez-Marí et al., 2010).

**Response:** In our analyses, compared with those in control females, the elevated expression of fancl in presumptive ovaries of cyp17a-/-;dmrt1-/- fish at 17, 23 and 25 dpf was positively correlated with its female-biased sex ratio. Actually, a moderated up-regulation of fancl in dmrt1-/- fish at 17-23 (Figure 2A and B) and 80 dpf (please see below, indicated with red box) was also observed by us.

However, it is not that significant as observed in the cyp17a1-/-;dmrt1-/- fish (Figure 2A and B). We interpret the highest up-regulation of fancl in cyp17a1-/-;dmrt1-/- fish to be the result of the compromised synergy of Dmrt1 and androgen signaling in inhibiting fancl transcription, as their synergistic effect in inhibiting the relative luciferase activity driven by fancl promoter (Figure 2H, Line 198-201).

Thanks for pointing the poor quality of the ISH on cryosection of immature gonads in our previous version of the manuscript. The new ISH on cryosections of immature gonads were run in triplicates during the manuscript was in revision. This could be supported by the previous study concerning the expression and function of fancl in zebrafish (Ref. 1). It was also observed that fancl was abundantly expressed in the follicles of cyp17a1-/-;dmrt1-/- fish than that of the control female fish (Figure 2C and D). On the other hand, the cDNA fragment of 786 nt containing the PHD domain of fancl was used to synthesize the antisense probe of fancl as previously described (Ref. 1). Furthermore, no obvious signal of NBT:BCIP staining could be observed when hybridized with the sense probe of fancl (Figure S1, Line 172-180).

**References:**

1. Rodriguez-Mari A, et al. Sex reversal in zebrafish fancl mutants is caused by Tp53-mediated germ cell apoptosis. PLoS Genet. 2010;6(7):e1001034.

5. This study and other reports showed the dysgenesis testis in some dmrt1-/- fish. The germ cell was lost in these mutants, as shown in Figure 4J. Why Vasa-positive germ cell was observed in the dmrt1-/- fish in Figure 5B? In the gonadal histology of cyp17a1-/-;dmrt1-/-;fancl-/- fish, no cystic germ cells was observed as shown in Figure 4L. However, it was observed by Vasa staining in Figure 5D. Whether mutation of tp53 can rescue the germ cell survival in dmrt1-/- and cyp17a1-/-;dmrt1-/-;fancl-/- fish?

**Response:** Thanks for pointing out this. Actually, based on our observations in previous Figure 5, germ cells were mostly depleted in dmrt1-/- male fish at 90 dpf, and the presence of few germ cells still could be observed in a low proportion of males (previous Figure 5B and D, green). The apoptotic germ cells were selected for statistical analysis and illustrated in previous Figure 5 only when its few Vasa-positive germ cells could be visualized in dmrt1-/- male fish and cyp17a1-/-;dmrt1-/-;fancl-/- fish; otherwise, it is difficult to distinguish the testis from other adipocyte tissues and impossible to detect the apoptotic of the germ cells.

The presence of the few Vasa-positive germ cells in dmrt1-/- male fish and cyp17a1-/-;dmrt1-/-;fancl-/- fish could be supported by the previous study of dmrt1-/- fish (Ref. 1,2), which possesses few to no germ cells. Even so, we agree that few germ cells of the testis adopted for the immunofluorescence assay in the previous version of our manuscript may be not the direct reflection of the dysgenesis testis with lost germ cells. To avoid confusing the readers, the previous Figure 5 has been given up.

Considering that Tp53 is a factor in the context of ovarian differentiation failure and not normal sex determination and differentiation, it would be exciting to know whether depletion of tp53 could prevent the all-testis differentiation in cyp17a1-/-;dmrt1-/-;fancl-/- fish. Of course, further efforts are required to obtain the cyp17a1-/-;dmrt1-/-;fancl-/-;tp53-/- fish (Line 347-350).

**References:**

1. Webster KA, et al. Dmrt1 is necessary for male sexual development in zebrafish. Developmental Biology. 2017;422:33-46.

2. Lin QH, et al. Distinct and cooperative roles of amh and dmrt1 in self-renewal and differentiation of male germ cells in zebrafish. Genetics. 2017;207(3):1007-22.

6. In Figure 3, the authors should demonstrate the protein interaction between TP53 and Fancl in vivo. Whether ubiquitination of TP53 showed difference in female and male gonads during zebrafish sex differentiation? Whether de-ubiquitination of TP53 can be detected in the fancl-/- fish? Fig 3D, the result of “IB: Myc” in the TCL group is missing.

**Response:** We agree that the in vivo evidence is more supportive to unravel the regulatory networks of different scenarios. Unfortunately, the appropriate antibody of Tp53 and Fancl is still not available. We have tried to verify several commercial antibodies of Tp53, but they did not work well in zebrafish to identify the target protein.

We understand that it should be clearly stated that these are interactions and activities as examined in HEK293T cells and the conclusions with respect to gonad development should be qualified to state that they are extrapolated from HEK293T cells to gonad. Therefore, the conclusions have been defined as “Assuming similar regulatory relationships exist in the germline and gonads” (Line 129, 318).

We apologize for the missing result of “IB: Myc” in the TCL group. This has been corrected in the current version of the manuscript (Figure 4C).

7. In Figure 5, no obvious TUNEL/Vasa co-location was presented. The authors should show high quality figures. Whether apoptosis was observed in the cyp17a1 mutants?

**Response:** As mentioned above, indeed, based on our observations in previous Figure 5, germ cells in dmrt1-/- male fish were mostly depleted at 90 dpf, and the presence of few germ cells could still be observed in a small proportion of males (previous Figure 5B and D, green). The apoptotic germ cells were selected for statistical analysis and shown in the previous Figure 5 only when the few Vasa-positive germ cells could be visualized in dmrt1-/- male fish and cyp17a1-/-;dmrt1-/-;fancl-/- fish, otherwise it is difficult to distinguish the testis from other adipocyte tissues and impossible to detect the apoptosis of the germ cells.

The presence of the few Vasa-positive germ cells in dmrt1-/- male fish and cyp17a1-/-;dmrt1-/-;fancl-/- fish could be supported by the previous study of dmrt1-/- fish (Ref. 1,2), which have few to no germ cells. Nevertheless, we agree that the few germ cells of the testis that were used for the immunofluorescence assay in the previous version of our manuscript may not be the direct reflection of the malformed testis with the loss of germ cells. The previous Figure 5 has been omitted in revised version to avoid reader confusion.

Meanwhile, based on our previous studies, the apoptosis was increased in the cyp17a1-deficient zebrafish and common carp (Ref. 3,4). The depletion of tp53 (IHB136, China Zebrafish Resource Center) did not affect sexual differentiation in zebrafish and could not restore all-testis differentiation in cyp17a1-/- zebrafish (in cyp17a1-/-;tp53-/- fish, N=12). This was also discussed in the current version of the manuscript (Line 343-347).

**References:**

1. Webster KA, et al. Dmrt1 is necessary for male sexual development in zebrafish. Developmental Biology. 2017;422:33-46.

2. Lin QH, et al. Distinct and cooperative roles of amh and dmrt1 in self-renewal and differentiation of male germ cells in Zebrafish. Genetics. 2017;207(3):1007-22.

3. Zhai G et al. Characterization of sexual trait development in cyp17a1-deficient zebrafish. Endocrinology. 2018;159(10):3549-62.

4. Zhai G et al. Successful production of an all-female common carp (Cyprinus carpio L.) population using cyp17a1-deficient neomale carps. Engineering. 2021;8,181-189.

8. Line 23-24, the sentence “Zebrafish has been used as a convenient model for teleost gonadal differentiation for many years” may not be necessary in abstract.

**Response:** According to your suggestion, this has been deleted in the revised abstract of the manuscript.

9. It should be noted that the sex reversal in mice are quite different from that in fish after cyp17a1 deletion. In mice, cyp17a1 deletion only resulted in underdeveloped accessory reproductive organs, leading to female-like external genitalia, the gonadal sex are not reversed. However, in fish, cyp17a1 deletion resulted in complete sex reversal. So, the results in mice cannot be used as evidence to support or discuss the role of androgen in gonadal differentiation or sex determination. The authors should consider to remove or adjust such description in Line 50, Line 68-70, Line 83, Line 381-383.

**Response:** We apologize for the inaccurate description. This has been corrected in the current version of the manuscript (Line 371-374).

10. It is well known that double mutation of cyp19a1a and dmrt1 resulted in ovary development in zebrafish and other fish species. In fact, the cyp19a1a;dmrt1 double mutants were equivalent to the cyp17a1;dmrt1 double mutants in absence of estrogen synthesis. Both are males due to estrogen deficiency. Therefore, the analyses of cyp17a1;dmrt1 double mutants did not provide us more information about fish sex differentiation. However, the different female rate in dmrt1-/-, cyp19a1a-/-;dmrt1-/- and cyp17a1-/-;dmrt1-/- zebrafish deserves more investigation.

**Response:** Thank you for your suggestion. For this comment, we would like to present as follows:

Though both the cyp17a1-/-;dmrt1-/- fish and cyp19a1a-/-;dmrt1-/- fish basically develop into males due to estrogen deficiency. However, as you point out that the ovarian appearance rates are different between these two mutants. This deserves more investigation. Both testis fates of cyp19a1a-/- fish and cyp17a1-/- fish can be basically reversed when dmrt1 was additionally depleted. However, the ovarian appearance rate in cyp19a1a-/-;dmrt1-/- fish is lower than the one in cyp17a1-/-;dmrt1-/- fish. The ratio of the ovary differentiation in cyp19a1a-/-;dmrt1-/- zebrafish decreases progressively from 60 to 100 dpf, with female ratios of 83%, 43% and 13% at 60, 75 and 100 dpf, respectively (Ref. 1). In contrast to this, the ratio of the ovarian appearance in cyp17a1-/-;dmrt1-/- fish at 90 dpf was 100% (Figure 1M).

Compared with the cyp19a1a-/-;dmrt1-/- fish, it is reasonable to speculate that the higher ratio of ovary differentiation displayed by cyp17a1-/-;dmrt1-/- fish might be attributed to its compromised androgen synthesis. Using previously published profiles, we compared endogenous steroid levels between cyp17a1-/- and cyp19a1a-/- fish. Testosterone was impaired and accumulated in cyp17a1-/- and cyp19a1a-/- fish, respectively (Ref. 2-4). Compared to cyp19a1a-/-;dmrt1-/- fish, it is reasonable to speculate that the higher ratio of ovarian differentiation in cyp17a1-/-;dmrt1-/- fish is due to impaired androgen synthesis. In other words, the synergistic effects of simultaneous depletion of Dmrt1 and androgen synthesis may exist in promoting gonadal embarkation on ovarian differentiation.

In other words, the synergistic effects of simultaneously depletion of dmrt1 and androgen synthesis may exist in promoting gonad embark upon ovary differentiation. This is in agreement with the ovary-biased differentiation phenotype reported in ar-/- zebrafish, which suggests that androgen signaling may indeed antagonize ovary differentiation in zebrafish (Ref. 3,5). Actually, a moderated up-regulation of fancl in dmrt1-/- fish at 17-23 and 80 dpf has been seen, which is not that significant as observed in cyp17a-/-;dmrt1-/- fish (please see our response to major comment 1). We interpret this phenotype to be the result of the synergistic effect of Dmrt1 and androgen signaling in inhibiting fancl transcription, as their synergistic effect in inhibiting the relative luciferase activity driven by fancl promoter (Line 198-201).

As you suggested, we have been investigating the difference between the female rate of dmrt1-/-, cyp19a1a-/-;dmrt1-/- and cyp17a1-/-;dmrt1-/- zebrafish (please see discussion section). The synergistic effects of Dmrt1 and androgen signaling antagonizing ovary differentiation in zebrafish might exist in zebrafish.

**References:**

1. Wu K, et al. Disruption of dmrt1 rescues the all-male phenotype of the cyp19a1a mutant in zebrafish - a novel insight into the roles of aromatase/estrogens in gonadal differentiation and early folliculogenesis. Development. 2020;147(4).

2. Zhai G, et al. Characterization of sexual trait development in cyp17a1-deficient zebrafish. Endocrinology. 2018;159(10):3549-62.

3. Yu G, et al. Zebrafish androgen receptor is required for spermatogenesis and maintenance of ovarian function. Oncotarget. 2018;9(36):24320-34.

4. Yin Y, et al. Targeted disruption of aromatase reveals dual functions of cyp19a1a during sex differentiation in zebrafish. Endocrinology. 2017;158(9):3030-41.

5. Crowder CM, et al. Nuclear androgen receptor regulates testes organization and oocyte maturation in zebrafish. Endocrinology. 2018;159(2):980-93.

11. The introduction should be refined according to the findings of this research to make the questions clear. In Line 110, “in some teleosts” should be revised as “in zebrafish”, because the references cited in this paragraph only studied zebrafish.

**Response:** The introduction has been thoroughly rewritten as suggested. The current order of the introduction includes sex determination in teleosts (Line 57-65), sex steroid synthesis and its relationship with dmrt1 (Line 66-90), the canonical theory of PGC numbers in determining sexual differentiation (Line 91-104), and the introduction to germ cell expressed fancl and its function (Line 105-125).

The “in some teleosts” has been revised as “in zebrafish” according to this suggestion (Line 95).

12. Three main findings of this study are: Dmrt1 antagonizes Fancl/TP53 signaling to regulate gonad differentiation in zebrafish without both estrogen and androgen; Fancl regulates the degradation of TP53 via K48 mediated polyubiquitination; Different female rate was observed between dmrt1-/- and cyp17a1-/-;dmrt1-/- zebrafish. The discussion needs to be re-structured according to the main findings of this study to make it more logical.

**Response:** We appreciate your valuable comments and suggestions. In our opinion, three main findings of this study are: synergistic effect of Dmrt1 and androgen signaling to antagonize Fancl/Tp53 signaling to regulate gonadal differentiation in zebrafish via inhibition of Fancl transcription (based on the different rate of ovary appearance observed between dmrt1-/- and cyp17a1-/-;dmrt1-/- zebrafish); regulatory role of Fancl in the degradation of Tp53 via K48-mediated polyubiquitination; insufficiency of accumulated endogenous progestin levels (in cyp17a1-/- fish) to maintain folliculogenesis in cyp17a1-/-;dmrt1-/- zebrafish (as for proper spermatogenesis in cyp17a1-/- fish). The discussion has been revised to incorporate all suggestions and to be more logical and concise.

13. The phenotype of cyp17a1-/-;dmrt1-/-;fancl-/- fish in this study was similar to that of the dmrt1-/-;rbmps2a-/-;rbmps2b-/- triple mutants. In zebrafish, maybe loss of genes related to germ cell development and survival results in the same phenotype. Please discuss it.

**Response:** Thank you for the good suggestion. This has been discussed in the current version of the manuscript (Line 328-339):

The severely hypoplastic testes observed in dmrt1-/-;fancl-/- fish and cyp17a1-/-;dmrt1-/-;fancl-/- fish are consistent with our previous view that Dmrt1 is required for the maintenance of male germ cells [42]. This could be interpreted that additional fancl depletion promotes testis differentiation in dmrt1-/- fish and cyp17a1-/-;dmrt1-/- fish, but their male germ cell development is dysregulated due to Dmrt1 deficiency. Similar observations have been reported with zebrafish whole testis differentiation upon depletion of RNA-binding protein of multiple splice forms 2 (rbmps2), a critical germline-expressed factor for female sex differentiation. The severe hypoplastic testes were observed in dmrt1-/-;rbmps2a-/-;rbmps2b-/- zebrafish [18]. Significantly, depletion of genes related to germ cell development and survival in fish results in the same phenotype of the lost germ cells as they attempt to embark on the male fate but suffer from the lack of Dmrt1 [18].

14. The scale bar in this manuscript should be adjusted to make it clear and consistent in each figure.

**Response:** Thanks for your suggestion. Accordingly, the images have been recaptured and the scale bars in Figures 1, 2, 3 and 6 have been updated. The oocytes at 50 and 90 dpf are shown with with a scale bar of 50 and 300 μm, respectively. Normal testes are shown with a scale bar of 25 μm, and the hypoplastic testes are shown with a scale bar of 10 μm (Line 410-412).
