# Supplementary material for: New insights into the all-testis differentiation in zebrafish with compromised endogenous androgen and estrogen synthesis
Source: PLoS Genet. 2024 Mar 7;20(3):e1011170. doi: 10.1371/journal.pgen.1011170 (PMC10919652; doi:10.1371/journal.pgen.1011170)
Supplement: Attachment — Submitted filename: 0 Response letter to P Genet 0124 .doc [file pgen.1011170.s012.doc]

Ms. Ref. No.: PGENETICS-D-23-01005R1

Title: New Insights into the All-testis Differentiation in Zebrafish with Compromised Endogenous Androgen and Estrogen Synthesis

**Dear Editor of *PLOS Genetics*,**

We appreciate the constructive comments of the reviewers! We have revised the manuscript according to the suggestions and addressed each of the reviewers' comments. A detailed point-by-point response to the reviewers' comments is provided. The comments are highlighted in blue, and our responses are given below.

According to the reviewers’ suggestions, the changes were made to the Abstract, Author Summary, Instructions, Results, Discussion, Figures and Materials and Methods. After these changes, we hope that our revised version of the manuscript is now acceptable for publication in your journal. We are also prepared to make further modifications if required.

Thank you very much for your consideration!

Zhan Yin

Ph. D.

Institute of Hydrobiology

Chinese Academy of Sciences

## *Response to Reviewer 1:*

**Reviewer #1:**

This revised manuscript from Ruan and colleagues investigates the factors regulating sex determination and differentiation in zebrafish. In this work the authors investigated the relationship between a conserved male differentiation factor, Dmrt1, and Cyp17a1a, a key enzyme in androgen and estrogen synthesis. In this revised version the authors have addressed the missing methods descriptions and statistical analyses. The authors have significantly revised the writing and added additional experiments to address concerns raised in the prior version; however, a few points, mostly related to writing, remain to be clarified.

**Response:** We appreciate the reviewer’s efforts in reviewing the manuscript and providing the constructive comments on the manuscript. We have revised our manuscript according to the suggestions point-by-point in the current version of the manuscript (please see below).

1. Allele designations are needed throughout the manuscript.

**Response:** Thanks for pointing out this. The designations of the allele mutants have been provided in the current version of the manuscript (New figure S1 and S3).

2. Line 192: The point is lost in the sentence beginning “on the other hand..” Stage of oocytes is an important factor here. If Fancl is more highly expressed in early oocytes and DM gonads are blocked in oocyte progression, there will be an enrichment in early oocyte expressed genes in DMs compared to SM or controls, thus expression would remain high. If fancl is less abundant in older oocytes this will appear as increased expression in double mutants since they will lack the later stage oocytes and genes expressed at later stages will be less abundant or absent in DMs. If fancl has multiple roles, its expression may be dynamic in oocytes - is this seen in in situ or in the published single cell RNAseq datasets? Also, if Dmrt1 represses fancl in oocytes, their expression patterns might be reciprocal in oocytes e.g. when Dmrt1 is high, fancl would be low... was this relationship analyzed in the scRNAseq datasets?

**Response:** This is an excellent suggestion for us to test whether Dmrt1 represses fancl in the oocyte and whether their expression patterns in the oocyte might be reciprocal. This is indeed interesting and could be done in oocytes of different stages in WT female zebrafish. However, for the reciprocal pattern of dmrt1 and fancl in oocytes, it could not determine in this study, because in cyp17a1-/-;dmrt1-/- fish, although a higher expression of fancl was observed, the dmrt1 itself is knocked out and its expression no longer useful (its function is lost). We are willing to make further efforts to carry out such a systemic work to dissect the transcriptional dynamics of these key genes involved in oocyte development and maturation, and their antagonistic effect.

In the absence of available data currently, we agree that the higher expression of fancl in cyp17a1-/-;dmrt1-/- fish at 80 dpf due to its high expression in primary oocytes or its lower abundance in growing and mature oocytes could not be excluded. This has been clarified in the current version of the manuscript, and this does not affect the conclusion (Line 187-190).

**References:**

1. Rodriguez-Mari A, et al. Sex reversal in zebrafish fancl mutants is caused by Tp53-mediated germ cell apoptosis. PLoS Genet. 2010;6(7):e1001034.

3. Line 230: The sentence beginning “There results suggest..” The wording here is not entirely consistent with the data. These results suggest that Fancl is required for ovary differentiation. While fancl might be a target, negatively regulated by Dmrt1, the data suggest that other Dmrt1 targets are required for testis development since the DM testis is infertile.

**Response:** Thanks for the suggestion. The sentence has been revised as “The results highlighting the antagonistic role of Fancl and Dmrt1 in determining gonadal sex, not only suggest that Fancl is required for ovary differentiation in dmrt1-/-;fancl-/- fish, but also imply the existence of other Dmrt1 targets required for testis development, as the testis is impaired in dmrt1-/-;fancl-/- fish” according to the suggestion (Line 217-220).

4. Line 278-280: a reference is needed for the sentence on progestin signaling.

**Response:** This has been revised according to the suggestion. The Ref 23 has been added (Line 269).

5) Line 309-310: “Indeed, we also observed a moderate up-regulation of fancl in dmrt1-/- fish at 17-23 dpf; however, it is not as significant as in cyp17a-/-;dmrt1-/- fish.” Can the possibility that this could be driven by differences in the stages of oocytes present in ovaries in these different mutant contexts be excluded? If not, please add a statement to qualify.

**Response:** Thanks for the suggestion. The statement has been revised as “This is unlikely to be driven by differences in the stages of oocytes present in the ovaries in our different mutant contexts, as the dmrt1-/- female zebrafish is fertile and develops normally [42]. The observed synergy between Dmrt1 and androgen signaling in inhibiting fancl transcription in luciferase reporter assays suggests that elevated fancl in cyp17a1-/-;dmrt1-/- fish may result from loss of these synergistic repressors” according to the suggestion (Line 294-299).

6. Line 342 and 343: Loss of Tp53 also did not rescue loss of Rbpms2 – Kaufman et al PLoS Genetics. Also, differentiation should be added along with cell survival and meiosis as failed differentiation will result in oocyte death.

**Response:** Thanks for the suggestion. The statement has been revised to “In contrast, introducing tp53 mutation did not restore ovary differentiation in dazl, figla, rbm46, vasa and rbpms2 mutants, which are key factors involved in germ cell survival, meiosis and differentiation [46-50]” according to the suggestion (Line 329).

7. In the “knockout lines” section of the methods, the guide RNA sequences and diagnostic primers should be provided. Alternatively, these sequences can be included in the supplemental table with the other primers.

**Response:** Thanks for the suggestion. The guide RNA sequences and diagnostic primers have been provided in revised Table 1 (Line 395-397, Table 1).

Minor writing:

1. Line 47-48: “With the all-testis phenotype observed in cyp17a1-/- zebrafish, it has been suggested that androgen signaling is dispensable for testis differentiation in zebrafish.” Consider instead: “The all-testis phenotype observed in cyp17a1-/- zebrafish, has led to the conclusion that androgen signaling is dispensable for testis differentiation in zebrafish”

**Response:** This has been revised according to the suggestion (Line 47-48).

1. Line 58: Instead of “entry into” consider “differentiation along”.

**Response:** This has been revised according to the suggestion (Line 57).

2. Line 59 and 60: “are complex due to its multiple types” Instead consider “are complex due to diversity within the species”

**Response:** This has been revised according to the suggestion (Line 58-59).

3. Line 66: Consider “influenced” rather than “enhanced”.

**Response:** This has been revised according to the suggestion (Line 65).

4. Line 77: “which” is not needed.

**Response:** This has been revised according to the suggestion (Line 76).

5. Line 79: “with” is not needed.

**Response:** This has been revised according to the suggestion (Line 77-78).

6. Lines 80-81: “Similarly these…” Consider instead “Similar phenotypes.”

**Response:** This has been revised according to the suggestion (Line 79-80).

7. Lines 93-95: The sentence beginning “It has been suggested that” should come later in the paragraph before the sentence on line 98 beginning “Dranow.”

**Response:** This has been revised according to the suggestion (Line 92, 96-97).

8. Line 99: Need to be specific and state that this is Nanos3 because mutants in the different nanos genes have different phenotypes.

**Response:** This has been revised according to the suggestion (Line 98).

9. Line 112: “in mutants with 12 of the 17 FA genes…” Instead consider, “ for 12 of the 17 FA mutants.

**Response:** This has been revised according to the suggestion (Line 111-112).

10. Line 116: Instead of “continue” consider “persist or continue to increase”.

**Response:** This has been revised according to the suggestion (Line 114-115).

11. Line 118: A linker or transition phrase would be helpful here." One factor known to mediate apoptosis in the developing gonad is Tp53".

**Response:** This has been revised according to the suggestion (Line 117-118).

12. Line 119: Required seems more appropriate than responsible here since Tp53 alone is probably not the sole factor.”

**Response:** Thanks for pointing out this. This has been revised according to the suggestion (Line 119).

13. Line 122: By “effectively” do you mean partially?

**Response:** Yes. This has been revised according to the suggestion (Line 121).

14. Line 127: By “with” do you mean "accompanied by", "associated with" or "along with"?

**Response:** Thanks for pointing out this. This has been revised according to the suggestion (Line 126).

15. Line 154: “a signal” or “signals” rather than “the signal”.

**Response:** This has been revised according to the suggestion (Line 153).

16. Line 154: “induce” rather than “exist in inducing”.

**Response:** This has been revised according to the suggestion (Line 153).

17. Line 157: “regulate” rather than “to be required for”.

**Response:** This has been revised according to the suggestion (Line 156).

18. Line 157: “of” is needed after differentiation.

**Response:** This has been revised according to the suggestion (Line 156).

19. Line 160: should indicate that this is in wildtype.

**Response:** This has been revised according to the suggestion (Line 159).

20. Line 160: “Based on their results…” this sentence is a bit unclear. Consider something like “Among these candidate genes fancl was selected based on its early expression in gonads at 17 and 23 dpf and its abundant expression in presumptive female gonads”.

**Response:** This has been revised according to the suggestion (Line 159-161).

21. Line 163: consider “aligns” rather than “correlates”.

**Response:** This has been revised according to the suggestion (Line 162).

22. Line 168: “the up-regulated..” Consider instead “Upregulation of fancl genes was observed...”

**Response:** This has been revised according to the suggestion (Line 166).

23. Line 170: “Moreover” rather than “besides”.

**Response:** This has been revised according to the suggestion (Line 168).

24. The sentence beginning, “To rule out the possibility…” There is a logic problem as written. It is not clear why a later change would be examined to control for a timing difference. The important issue is the ability to compare the stages present in the gonad to exclude the possibility that the expression difference is due to a difference in stages/types of cells present withing the gonads. Based on the images shown, it appears that there may be more early-stage cells present in the cyp17;dmrt double mutants compared to controls. To determine if increased expression is due to upregulation or more cells expressing, cells of the same stage should be compared.

**Response:** We apologize for providing the unclear details. This has been revised in the current version of the manuscript (Line 170-172).

Compared to the higher expression of fancl in cyp17a1-/-;dmrt1-/- fish at the juvenile stage, the reviewer suggested that the increased expression of fancl may be due to more fancl-expressing cells at early stage. For this issue, we would like to present as follows:

In the absence of currently available data, we agree that the higher expression of fancl in cyp17a1-/-;dmrt1-/- fish at 80 dpf may be due to its high expression in primary oocytes or its lower abundance in growing and mature oocytes (please see response to comment 2 and Line 187-190). However, it should be noted that, in addition to the higher expression of fancl in the gonads of fish at 25 dpf, it was also observed in cyp17a1-/-;dmrt1-/- fish at 17 and at 23 dpf, a stage in which the gonads were still undifferentiated (Figure 2A and B) (Ref. 1). More importantly, among the different timepoints examined, the cyp17a1-/-;dmrt1-/- fish showed a higher increase in fancl expression at 17 dpf (2.39-fold, Figure 2A) than 23 dpf (1.63-fold, Figure 2B). Combined with the expression characteristics of fancl in cyp17a1-/-;dmrt1-/- fish at 17, 23, 25 and 80 dpf and the fancl:luciferase assay analysis, it is convincing to link the sex determination phenotypes observed in cyp17a1-/-, dmrt1-/- and cyp17a1-/-;dmrt1-/- fish to the induction of fancl expression.

**References:**

1. Rodriguez-Mari A, et al. Sex reversal in zebrafish fancl mutants is caused by Tp53-mediated germ cell apoptosis. PLoS Genet. 2010;6(7):e1001034.

25. Line 178 and elsewhere: Consider greater, higher, or more abundant rather than “upregulated” because expression could be higher due to increased expression (upregulation) or stability.

**Response:** This has been revised according to the suggestion (Line 28, 155, 174, 197, 199, 224).

26. Line 180: “but exhibited no obvious signals of staining..” Consider instead, “and as expected no signals were detected”.

**Response:** This has been revised according to the suggestion (Line 176).

27. Line 181: “The” is not needed before comparative, and “in ovaries” should be “between ovaries”.

**Response:** This has been revised according to the suggestion (Line 177).

28. Line 182: “the” is not needed before “cyp17a1” and “fish of their” is not needed between female and control.

**Response:** This has been revised according to the suggestion (Line 178).

29. Line 184 “alterations of the expression levels..” Consider instead “expression level alterations of 424 genes”.

**Response:** This has been revised according to the suggestion (Line 180).

30. Line 186: “The majority of KEGG..” Consider instead “the most enriched KEGG pathways in..”

**Response:** This has been revised according to the suggestion (Line 181).

31. Line 220: The sentence beginning “However” is unclear. Do you mean “Unlike cyp;dmrt1 DMs which developed ovaries, cyp;dmrtq;fancl TMs developed testis with histologically apparent abnormalities including fb like somatic cells.....similar to those observed in...”

**Response:** Yes. Thanks for the suggestion and this has been revised in the current version of the manuscript (Line 208-211).

32. Line 224: The sentence beginning “The dissected testes..” can be simplified. “Accordingly, dissected testis of SM and TM were hypoplastic compared to controls.” Details about fixative should be in the methods section.

**Response:** Thanks for the suggestion. This has been revised in the current version of the manuscript (Line 213-214).

33. Line 228: “also has a hypoplastic testis…” Consider instead “testes were also hypoplastic and lack germ cells similar to dmrt1-/-“.

**Response:** This has been revised according to the suggestion (Line 215-216).

34. Line 237: “which is an all-ovary differentiation mechanism..” Should be "context" rather than "mechanism".

**Response:** This has been revised according to the suggestion (Line 225).

35. Line 247: “mutant domain” should be “functional domain” unless the activity of the domain was altered.

**Response:** This has been revised according to the suggestion (Line 235).

36. Line 262: “albeit the” should be deleted.

**Response:** This has been revised according to the suggestion (Line 250).

37. Line 262 “the additional supplement..” consider instead “supplementation with estrogen..”

**Response:** This has been revised according to the suggestion (Line 250).

38. Line 265 “the” is not needed before treatment and “of” should be “with”

**Response:** This has been revised according to the suggestion (Line 252).

39. Line 266: “ovary” should be “ovaries”.

**Response:** This has been revised according to the suggestion (Line 253).

40. Line 267: “follicles of the” rather than “that of the”.

**Response:** This has been revised according to the suggestion (Line 254).

41. Line 268: “were” rather than “was” and “at PG..” rather than “in the PG”.

**Response:** This has been revised according to the suggestion (Line 255).

42. Line 268: “stages” rather than “stage”.

**Response:** This has been revised according to the suggestion (Line 255).

43. Line 275: “the” is not needed before cyp17a1

**Response:** This has been revised according to the suggestion (Line 261).

44. Line 277: “albeit” should be deleted.

**Response:** This has been revised according to the suggestion (Line 263).

45. Line 278: “The” is not needed before augmentation.

**Response:** This has been revised according to the suggestion (Line 263).

46. Line 278: Consider “proposed” rather than “found”.

**Response:** This has been revised according to the suggestion (Line 264).

47. Lines 280-282: This section is somewhat confusing as written. If supplementing with Progestin supports testis organization, then it makes sense that the receptor would be required for normal testis development. This result suggests progestin is important for normal organization of the testis and spermatogenesis, but not for determination of testis fate.

**Response:** Thanks for the suggestion. This has been revised in the current version of the manuscript (Line 267-269).

48. Line 286-287: By “an interactive function” do the authors mean antagonistic rather than interactive.

**Response:** Thanks for the suggestion. This has been revised in the current version of the manuscript (Line 271).

49. Line 289: “despite” rather than “albeit”.

**Response:** This has been revised according to the suggestion (Line 274).

50. Line 290: “the” is not needed before ovarian differentiation. In the same sentence, the data suggest this would be maintenance rather than ovary differentiation if ovaries are found among mutants prior to d60 but decline thereafter.

**Response:** Thanks for the suggestions. This has been revised in the current version of the manuscript (Line 275-276).

51. Line 293: “the ovarian appearance..” Consider instead “ovaries detected among”..

**Response:** This has been revised according to the suggestion (Line 278).

52. Line 297: “elevated” rather than “accumulated”.

**Response:** This has been revised according to the suggestion (Line 281).

53. Line 298: “incidence or frequency” rather than “rate”.

**Response:** This has been revised according to the suggestion (Line 283).

54. Line 300-301: “may exist in promoting gonadal embarkation on ovarian differentiation.” Consider instead “may promote ovary differentiation”.

**Response:** This has been revised according to the suggestion (Line 285).

55. Line 310: The sentence beginning “The highest up regulation of fancl” can be simplified “Elevated fancl..” Later in the same sentence starting on line 312 “, as their synergistic effect in inhibiting the relative” can be deleted. This sentence would be clearer if these thoughts were inverted. "The observed synergy between Dmrt1 and Androgen signaling in inhibiting fancl transcription in luciferase reporter assays suggests that elevated fancl in cyp17;dmrt1 DM may result from loss of these synergistic repressors.”

**Response:** Thanks for the suggestions. This has been revised in the current version of the manuscript (Line 294-297).

56. It would help to start the paragraph starting on line 315 with a broad sentence regarding cell death/oocyte survival and implicated pathways to introduce Tp53 which otherwise appears out of the blue.

**Response:** This has been revised according to the suggestion (Line 300-302).

57. Line 325 “it could not maintain ovary differentiation” can be simplified “due to impaired ovary differentiation”.

**Response:** This has been revised according to the suggestion (Line 311).

58. Line 326 “germ cell survival”. Do the authors mean oocyte? or are the authors proposing that Fancl might regulated GC survival in ovary and testis?

**Response:** Disruption of fancl is known to cause masculinized gonads and testis differentiation due to increased germ cell apoptosis compromises oocyte survival, which could be rescued by tp53-depletion (Ref.1). In this study, additional depletion of fancl also resulted in all-testes differentiation in cyp17a1-/-;dmrt1-/- fish, due to impaired ovary differentiation. Together with the genetic data and molecular biology assays, we propose that the Fancl-mediated germ cell survival is determinant in gonadal differentiation in cyp17a1-/-;dmrt1-/- zebrafish. This has been discussed in the current version of the manuscript (Line 300-302, 311-313).

**References:**

1. Rodriguez-Mari A, et al. Sex reversal in zebrafish fancl mutants is caused by Tp53-mediated germ cell apoptosis. PLoS Genet. 2010;6(7):e1001034.

59. Line 345-346: “(in cyp17a1-/- ;tp53-/- fish, N=12).” This should be in the results section not the discussion.

**Response:** This has been revised according to the suggestion (Line 329-332).

60. Line 351: “maintains proper” rather than “properly maintains”.

**Response:** This has been revised according to the suggestion (Line 337).

61. Line 365: “elevated” rather than “accumulated”.

**Response:** This has been revised according to the suggestion (Line 351).

62. Line 369: “The” is not needed before determination.

**Response:** This has been revised according to the suggestion (Line 355).

63. Line 372: “the” is not needed before depletion.

**Response:** This has been revised according to the suggestion (Line 358).

64. Line 372: “for” is needed before “CYP17A1”.

**Response:** This has been revised according to the suggestion (Line 358).

65. Line 389: “respectively” is not needed.

**Response:** This has been revised according to the suggestion (Line 375).

66. Line 394 and 396: “used” rather than “adopted”.

**Response:** This has been revised according to the suggestion (Line 386, 388).

## *Response to Reviewer 2:*

**Reviewer #2:**

The authors have addressed all of my previous concerns and I have no further concerns.

**Response:** We appreciate the reviewer’s efforts in reviewing the manuscript and providing the constructive comments on the manuscript.

## *Response to Reviewer 3:*

**Reviewer #3:**

The authors have answered almost all my questions. The writing of the current manuscript becomes more readable and friendlier for readers. I think it would be better if the following suggestion can be considered before publication.

**Response:** We appreciate the reviewer’s efforts in reviewing the manuscript and providing the constructive comments on the manuscript. We have revised our manuscript according to the suggestions point-by-point in the current version of the manuscript (please see below).

Abstract

1. Line 31-32: The description “The all-ovary differentiation phenotype observed in cyp17a1-/-;dmrt1-/- zebrafish can be rescued by additional depletion of fancl” feels like that the gonads of cyp17a1-/-;dmrt1-/-;fancl-/- fish will develop into all-ovary”.

**Response:** Thanks for the suggestion. The statement has been revised as “The additional depletion of fancl in cyp17a1-/-;dmrt1-/- zebrafish reversed the gonadal sex differentiation from all-ovary to all-testis (in cyp17a1-/-;dmrt1-/-;fancl-/- fish)” according to the suggestion (Line 30-32).

2. Line 26-32: In the present study, the female-biased sex ratio phenotypes were positively correlated with upregulated levels of Fanconi anemia complementation group L (fancl) in the gonads of doublesex and mab-3 related transcription factor 1 (dmrt1)-/- and cyp17a1-/-;dmrt1-/- fish, as fancl transcription being synergistically inhibited by Dmrt1 and androgen signaling. The all-ovary differentiation phenotype observed in cyp17a1-/-;dmrt1-/- zebrafish can be rescued by additional depletion of fancl”. Is it possible to write in the following way: “In the present study, the female-biased sex ratio phenotypes......, and additional depletion of fancl in cyp17a1-/-;dmrt1-/- zebrafish reversed the female-biased sex ratio to...... Luciferase assay revealed synergistic inhibitory effect of Dmrt1 and androgen signaling to fancl transcription”. “as fancl transcription being synergistically inhibited by Dmrt1 and androgen signaling” should be “as fancl transcription is/was synergistically inhibited by Dmrt1 and androgen signaling”

**Response:** We appreciate the suggestion. This section has been re-written according to this suggestion (Line 26-33).

3. Line 33: Change “Tumor Protein p53” into “Tumor protein p53”.

**Response:** This has been revised according to the suggestion (Line 34).

Author summary

4. Line 53-55: “Our current study provides new insights into the interactive signals that regulate sexual fate determination with impaired androgen and estrogen synthesis in teleosts”. It would be better to remove the description “with impaired androgen and estrogen synthesis”.

**Response:** This has been revised according to the suggestion (Line 53-54).

Introduction

5. Line 67: Change “including” to “especially”.

**Response:** This has been revised according to the suggestion (Line 66).

Results

6. Line 205-213: “cyp17a1-/-;dmrt1-/- fish were derived...... then inbred to generate triple homozygotes (cyp17a1-/-;dmrt1-/-;fancl-/-)”. I think this part can be moved to Materials and methods section.

**Response:** This has been revised according to the suggestion (Line 200-202).

7. Line 231-232: “strengthen the notion that fancl is the downstream regulator of dmrt1 in determining testis fate”. Maybe it is not suitable to say that fancl is the downstream regulator of dmrt1 in testis fate determination. Is fancl expressed in testis? Is fancl downstream of dmrt1 in testis? Maybe it can be written as “highlighting the antagonistic role of fancl and dmrt1 in determining gonad sex”.

**Response:** This has been revised according to the suggestion. The statement has been revised as “The results highlighting the antagonistic role of Fancl and Dmrt1 in determining gonadal sex” (Line 217-218).

Figures

8. Figure 2C, D: Please quantify the increased expression observed for fancl.

**Response:** Good suggestion. The expression of fancl by in situ hybridization in presumptive ovaries from control fish and cyp17a1-/-;dmrt1-/- fish at 25 dpf has been quantified according to this suggestion (New Figure 2E, Line 412-415).

Figure legends

9. Line 840: “90 dpf” should be “110 dpf”.

**Response:** This has been revised according to the suggestion (Line 792).
